# Comparison of Genetic Diversity between Chinese and American Soybean (*Glycine max* (L.)) Accessions Revealed by High-Density SNPs

**DOI:** 10.3389/fpls.2017.02014

**Published:** 2017-11-30

**Authors:** Zhangxiong Liu, Huihui Li, Zixiang Wen, Xuhong Fan, Yinghui Li, Rongxia Guan, Yong Guo, Shuming Wang, Dechun Wang, Lijuan Qiu

**Affiliations:** ^1^National Key Facility for Gene Resources and Genetic Improvement, Key Laboratory of Crop Germplasm Utilization, Ministry of Agriculture, Institute of Crop Sciences, Chinese Academy of Agricultural Science, Beijing, China; ^2^Department of Plant, Soil and Microbial Sciences, Michigan State University, East Lansing, MI, United States; ^3^Institute of Soybean Research, Jilin Academy of Agricultural Sciences, Changchun, China

**Keywords:** soybean, single nucleotide polymorphism (SNP), genetic diversity, population structure, US soybean accessions, Chinese soybean accessions

## Abstract

Soybean is one of the most important economic crops for both China and the United States (US). The exchange of germplasm between these two countries has long been active. In order to investigate genetic relationships between Chinese and US soybean germplasm, 277 Chinese soybean accessions and 300 US soybean accessions from geographically diverse regions were analyzed using 5,361 SNP markers. The genetic diversity and the polymorphism information content (PIC) of the Chinese accessions was higher than that of the US accessions. Population structure analysis, principal component analysis, and cluster analysis all showed that the genetic basis of Chinese soybeans is distinct from that of the USA. The groupings observed in clustering analysis reflected the geographical origins of the accessions; this conclusion was validated with both genetic distance analysis and relative kinship analysis. *F*_ST_-based and EigenGWAS statistical analysis revealed high genetic variation between the two subpopulations. Analysis of the 10 loci with the strongest selection signals showed that many loci were located in chromosome regions that have previously been identified as quantitative trait loci (QTL) associated with environmental-adaptation-related and yield-related traits. The pattern of diversity among the American and Chinese accessions should help breeders to select appropriate parental accessions to enhance the performance of future soybean cultivars.

## Introduction

Soybean originated in China, and has a history of planting for more than 4,000 years (Hymowitz and Newell, 1981). It was introduced to the USA in 1765 as animal feed firstly (Hymowitz and Harlan, 1983) and has been widely planted in the USA since 1922. To date, soybean is one of the most important economic crops for both China and the USA.

There are extensive soybean breeding programs in both China and the USA, most of which rely on a genetic base of Chinese origin (Cui et al., 2001). There have been over 400 publicly released cultivars in the USA, derived from ~80 soybean ancestral lines (Gizlice et al., 1994), most of which were originally introduced from China in the early twentieth century (Li et al., 2001). In addition, during past decades, the exchange and utilization of soybean germplasm between the two countries has been and continues to be active. The Chinese Gene Bank contains 28,580 soybean accessions, of which 1,718 (6.01%) were introduced from the USA. There are 14,330 soybean germplasm accessions that are conserved in USDA Soybean Germplasm Collection in the USA, of which 5,216 (36.40%) are from China. Some elite cultivars from North America were used in Chinese soybean breeding programs for broadening the genetic base of modern soybean cultivars. During the period from 1923 to 2005, 1,300 soybean cultivars were released in China; according to a study of the pedigree of these cultivars, the genetic contribution from US cultivars to Chinese cultivars was 139.83, accounting for 10.76% of the total (Gai et al., 2015). Moreover, Carter et al. (2000) suggested that Chinese cultivars should also be viewed as an important reservoir of genetic diversity that can be used to yet further expand the genetic base for North American soybean breeding efforts. Therefore, considering the ongoing increases in exchange and utilization of soybean germplasm between the two countries, assessing genetic relationships is a worthy area for further study.

The pattern of genetic variation in soybean germplasm resources between China and USA has been evaluated through pedigree information (Cui et al., 2000a,b), phenotypic observation (Cui et al., 2001), and low-density polymorphism markers (Qiu et al., 1997; Carter et al., 2000; Li et al., 2001). Gizlice et al. (1994) analyzed the pedigrees of 258 publicly developed cultivars in US released between 1947 and 1988, and determined that >84% of their parentage could be traced back to only 17 ancestors, and most of which were selected from the introduced landraces from China. Based on the pedigree analysis, Cui et al. (2000a,b) found that accessions from the two countries had few identifiable ancestors in common probably because of different environments and breeders' selection preference between two countries. Using 25 biochemical, morphological, and agronomic traits, Cui et al. (2001) found much more extensive phenotypic diversity among the Chinese cultivars than among North America cultivars, with obvious distinctness between the two groups. Using evaluation based on random amplified polymorphic DNA (RAPD) markers, Li et al. (2001) demonstrated that the ancestors of soybean cultivars from North America and China were clearly different. To date, previous reported results in this research area have been based on a relatively small number of progenitor soybean cultivars using relative low-density molecular markers.

Significant progress has been made using high throughput genotyping technologies to detect variability in DNA sequences, and these technologies are now used regularly in crop germplasm research and breeding (Akond et al., 2013; Li et al., 2014; Lee et al., 2015; Zhou et al., 2015, 2016; Han et al., 2016; Wang et al., 2016; Chang and Hartman, 2017; Fang et al., 2017; Liu et al., 2017a). In the present study, a total of 577 soybean cultivars or advanced breeding lines from the main production areas of China and the USA were analyzed using the 5,361 SNP markers on the Illumina SoySNP6k iSelect BeadChip. The objectives were (i) to compare the genetic diversity between the American and Chinese soybeans; (ii) to investigate the population structures for these subpopulations; and (iii) to identify loci that have undergone selection based on differences in allele frequencies between the subpopulations. The results in present study will provide useful information to future soybean breeding for both China and the USA.

## Materials and methods

### Plant materials

**Figure 1:**
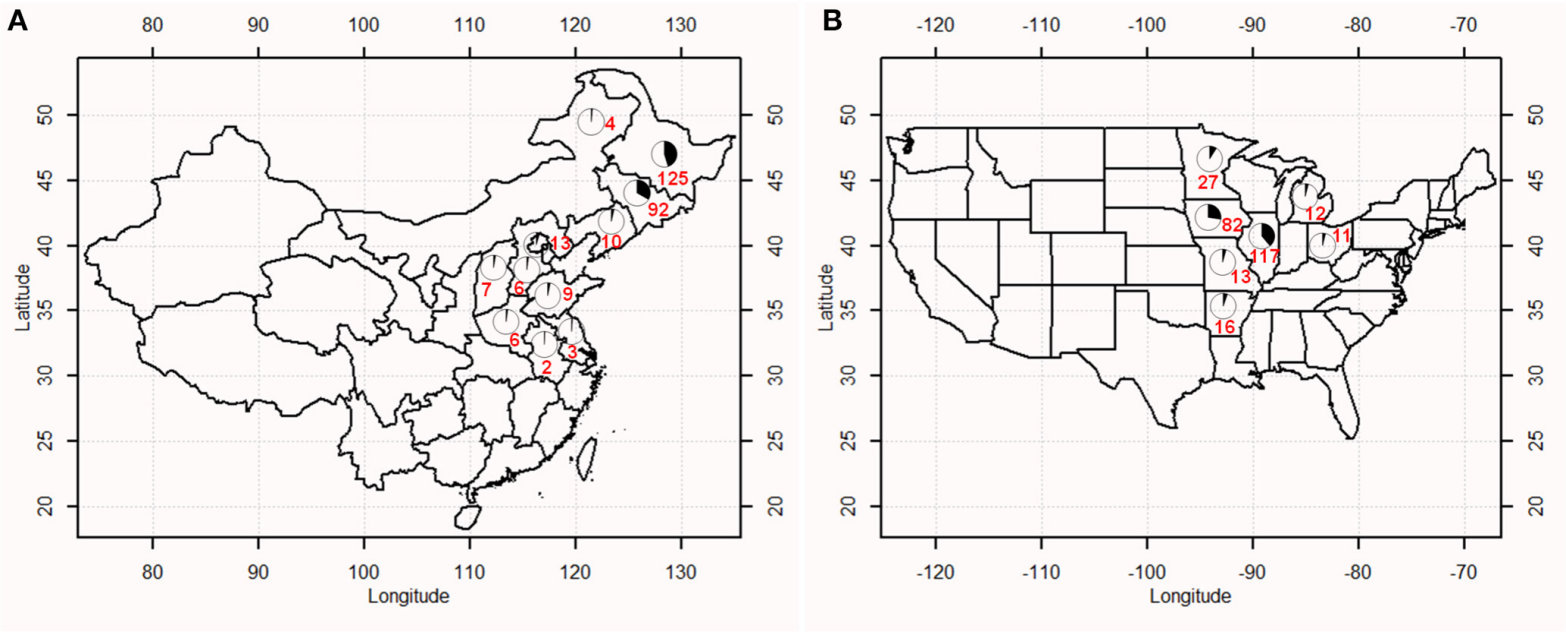
Geographic distribution of soybean accessions in China **(A)** and the USA **(B)**. Numbers marked on the map indicated the number of accessions from a given region.

This study examined soybean cultivars and advanced breeding lines: 277 from China (hereafter termed as CN-set) and 300 from the USA (hereafter termed as US-set). Of the 277 Chinese accessions collected from 11 provinces, 231 accessions were derived from the Northern spring (Nsp) ecotype and 46 were derived from the Huang-huai-hai summer (Hsu) ecotype. Detailed information on the 277 accessions can be found in Supplementary Table S1. The 300 diverse accessions of US-set represent a range of materials developed by public breeders in the North Central soybean production area of the USA (Wen et al., 2014); detailed information on these 300 accessions was listed in Supplementary Table S2. The geographic distribution of the 577 soybean accessions was presented in Figure 1.

### DNA extraction and SNP genotyping

Genomic DNA was extracted from soybean seedlings (leaf) following the protocol presented by Kisha et al. (1997). All of the accessions in the present study were genotyped with the Illumina SoySNP6k iSelect BeadChip (Illumina, USA), which can be used to distinguish different genotypes among 5,361 SNPs (Akond et al., 2013); the chromosomal distributions and quality control for these SNPs was demonstrated in Wen et al. (2014).

### Basic population genetic parameters

Using software of PowerMarker 3.25 (Liu and Muse, 2005), minor allele frequency (MAF), genetic diversity, polymorphism information content (PIC), and heterozygosity, were evaluated for each population pool (i.e., the CN-set or the US-set) across the soybean genome. The average and the range of the Roger distance within and between subpopulations, and across all genotypes, were calculated. The degree of linkage disequilibrium (LD) was evaluated by LD parameter *r*^2^, calculated by TASSEL 5.0 (Bradbury et al., 2007). The decay distance of LD at *r*^2^ = 0.1 was determined as the length of a LD block. Kinship matrix analysis (Loiselle et al., 1995) in TASSEL 5.0 was conducted to uncover the genetic identity between two given accessions by $K_{ij}=\sum_{l}\left[\sum_{a}(p_{ila}-p_{la})(p_{jla}-p_{la})+\sum_{a}\frac{p_{la}(1-p_{la})}{\left(n_{l}-1\right)}\right]/\sum_{l}\sum_{a}\left(p_{la}\left(1-p_{la}\right)\right),$ where *p*_*ila*_ is the frequency of allele *a* at locus *l* in individual *i*; *p*_*la*_ is the frequency of allele *a* at locus *l* in the reference sample; *n*_l_ is the number of alleles defined in the sample at locus *l* (the number of individuals times the ploidy level minus the number missing alleles); and (*n*_l_ − 1) is a sampling bias correction. Negative values between two accessions, indicating the existence of a weaker relationship than would be expected between two random individuals, were replaced by zero. Analysis of molecular variance (AMOVA) was performed to estimate the variance between populations and among accessions within populations based on analyses of variance of allele frequencies (Excoffier et al., 1992) using Arlequin 3.5 software (Excoffier and Lischer, 2010). The population fixation statistic *F*_ST_ between the CN-set and US-set was calculated genome-widely using Arlequin 3.5.

### Population structure analysis

Three multivariate analyses, including model-based population structure analysis, principal component analysis (PCA), and cluster analysis with a neighbor-joining algorithm, were employed to divide the soybean accessions into subgroups. The Bayesian model-based program STRUCTURE 2.3 (Pritchard et al., 2000) was used to infer the population structure and to assign the 577 genotypes into subpopulations based on 5,195 polymorphic SNP markers (there were 166 SNPs of the 5,361 SNPs on the Illumina SoySNP6k iSelect BeadChip for which no data points were obtained in more than 20% of the accessions; see the first section of the results). Ten independent analysis instances, based on 100,000 MCMC replications and 100,000 burn-ins, were performed, with the hypothetical number of subpopulations (*k*) ranging from 1 to 10. The number of subpopulations was determined when Δ*k* reached its highest value (an *ad hoc* statistic; Evanno et al., 2005). PCA and cluster analysis were implemented in TASSEL 5.0.

### Identification of loci under selection

Three statistical methods were used to detect the loci under selection. (1) Differences in allele frequency between the CN-set and the US-set were tested by Student's *t*-test: $t=\frac{f_{1}-\;f_{2}}{\sqrt{\left(\frac{1}{2n_{1}}\;+\;\frac{1}{2n_{2}}\right)f_{exp}\left(1\;-\;f_{exp}\right)}}$, where $f_{exp}=\frac{f_{1}n_{1}\;+\;f_{2}n_{2}}{n_{1}\;+\;n_{2}}$, *f*_1_ and *f*_2_ were the allele frequencies in the CN-set and US-set, respectively, and *n*_1_ and *n*_2_ were the sample sizes in the CN-set and US-set, respectively. The population-specific alleles were determined for each subpopulation based on zero allele frequency in one subpopulation and non-zero in another subpopulation; and different allele frequency between two subpopulations reaching at significance level *P* < 0.001. (2) *F*_ST_ between the CN-set and US-set was calculated for individual SNP using VCFtools (https://vcftools.github.io/index.html) (Weir and Cockerham, 1984) and EigenGWAS (Chen et al., 2016). The VCFtools was conducted with a sliding window of 100 kb and a step size of 10 kb (Schmutz et al., 2014) over the whole genome, and the regions with the top 5% of *F*_ST_-values were regarded as highly diverged across the two groups. (3) Finding loci under selection through genome-wide association studies of eigenvectors were implemented by EigenGWAS. There were three steps included. Firstly, genetic relationship matrix was generated for the 577 accessions; secondly, the top 10 eigenvalues and eigenvectors were calculated; and then linear model for selected eigenvectors from the second step was conducted.

## Results

### Genetic diversity

**Figure 2:**
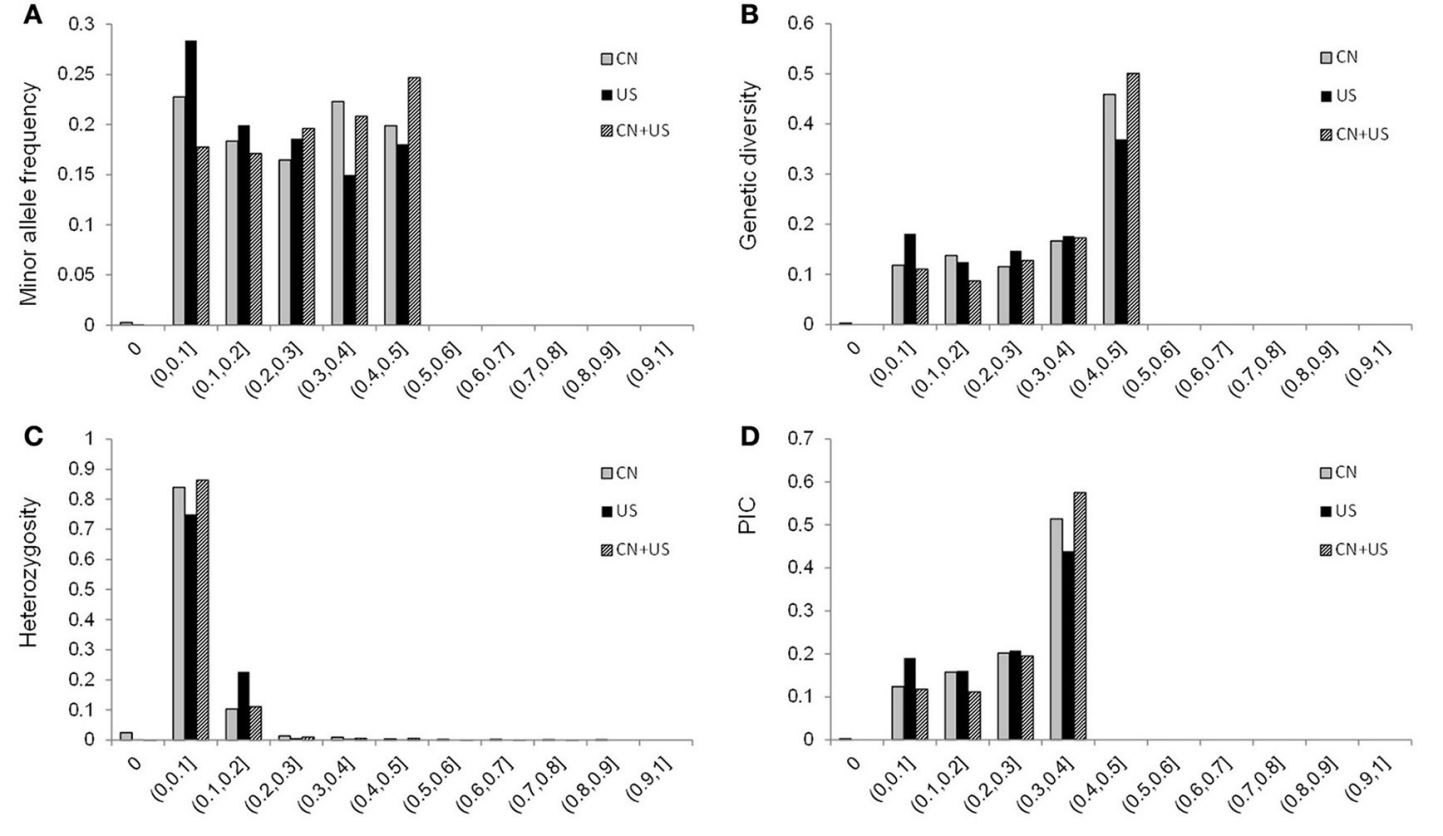
Distribution of the minor allele frequency (MAF; **A**), genetic diversity **(B)**, heterozygosity **(C)**, and PIC **(D)** of 5,195 SNPs across 577 accessions combined, for 277 accessions from China, and for 300 accessions from the USA. CN is for China; US is for United States of America; and CN+US is for China and US accessions analyzed together.

A total of 577 soybean accessions were analyzed using the 5,361 SNP markers of the Illumina SoySNP6k iSelect BeadChip. SNP markers with missing data points for more than 20% (166 SNPs) of the accessions were not used for further analysis, so a total of 5,195 SNPs (96.90%) were used in our study. In the CN-set, the average PIC was 0.2643, ranging from 0 to 0.3750, and the average genetic diversity was 0.3307, ranging from 0 to 0.5000. In the US-set, the PIC ranged from 0 to 0.3750, with an average of 0.2408, and genetic diversity ranged from 0 to 0.5000, with an average of 0.2988 (Table 1 and Figure 2). These results indicated that the Chinese soybean accessions had a higher level of genetic diversity than the US soybean accessions. As expected, there was an increase in the estimates of PIC and genetic diversity for the entire diversity collection (Table 1 and Figure 2), as compared to either the CN-set or the US-set.

**Table 1:** Genetic parameters revealed by the analysis of 5,195 polymorphic SNP markers among the soybean accessions of the diversity panel.

**Origin**	**Minor allele frequency**	**Genetic diversity**	**Heterozygosity**	**PIC**
CN	0.2498 (0~0.5000)	0.3307 (0~0.5000)	0.0658 (0~0.8654)	0.2643 (0~0.3750)
US	0.2209 (0~0.5000)	0.2988 (0~0.5000)	0.0762 (0~0.9588)	0.2408 (0~0.3750)
CN+US	0.2679 (0.0026~0.5000)	0.3489 (0.0052~0.5000)	0.0711 (0~0.7750)	0.2769 (0.0052~0.3750)

*CN is for China; and US is for United States of America*.

**Figure 3:**
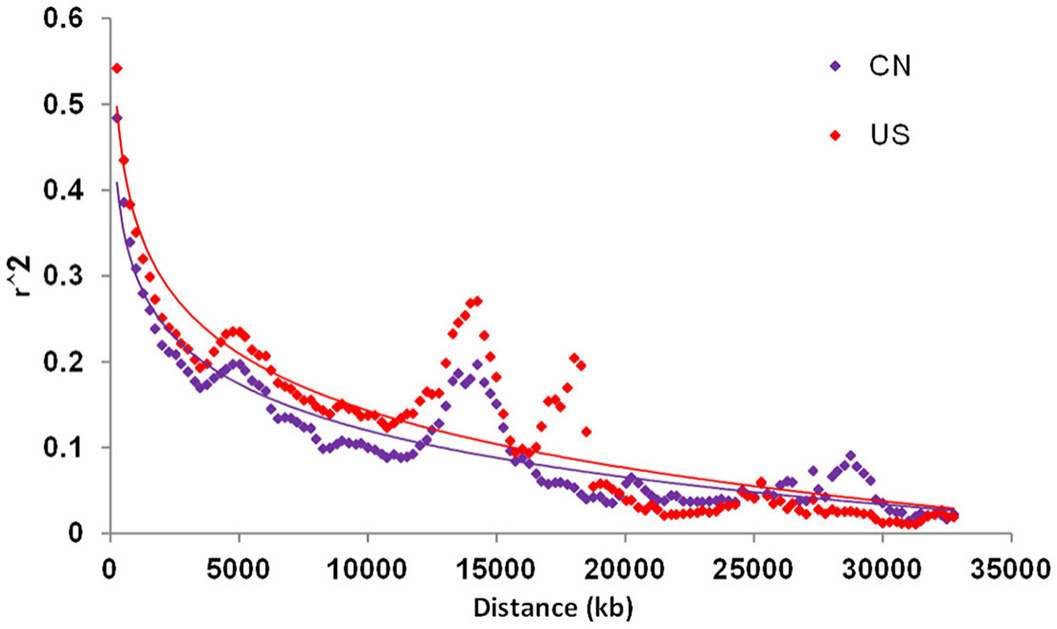
Linkage disequilibrium (LD) decay of China and US soybean accessions. CN is for China; and US is for United States of America.

Linkage disequilibrium (LD) analysis was performed based on 5,195 SNPs in both the CN-set and the US-set. The *r*^2^ statistic was calculated and tested for each pairwise SNP to measure the degree of LD (Figure 3). In the CN-set, we found that *r*^2^ for 58.41% of the pairwise SNPs were significant level at the alpha = 0.01. Comparatively, in the US-set, *r*^2^ for 59.96% of the pairwise SNPs were significant level at the alpha = 0.01. In both sets, LD gradually declined with increased physical distance. The distance over which LD decays to *r*^2^ = 0.1 was 8,500 kb in the CN-set and 15,500 kb in the US-set. Thus, our LD analysis also indicated that the Chinese soybeans have a higher level of genetic diversity than the American soybeans.

### Unbiased population structure analysis

**Figure 4:**
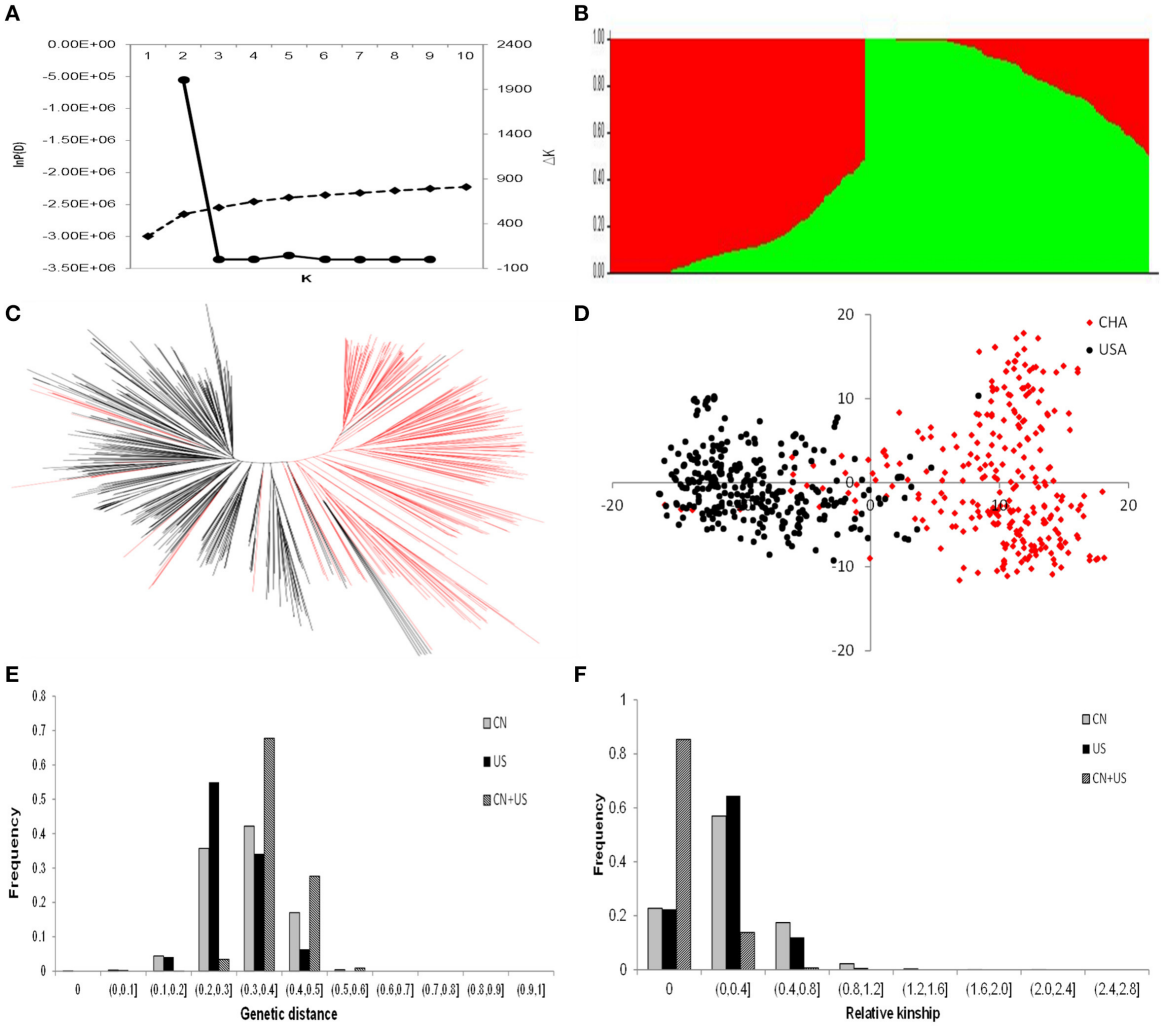
Genetic structure and relatedness of populations from China and America. **(A)** Evolution of the natural logarithm probability of the data against *K* and the magnitude of Δ*K* for each *K-*value; **(B)** Clustering for *K* = 2 for the entire set of soybean accessions. Each individual is represented by a vertical bar, partitioned into colored segments with the length of each segment representing the proportion of the individual's genome from groups when *k* = 2; **(C)** Neighbor-joining tree constructed using SNP data; **(D)** Principal component analysis for the entire set of soybean accessions. Soybean accessions from China are shown in red, and soybean accessions from the United States of America are shown in black; **(E)** Distribution of the genetic distance for 277 Chinese and 300 American soybean accessions; and **(F)** Distribution of the kinship values for 277 Chinese and 300 American soybean accessions. CN is for China; and US is for United States of America.

We investigated the possible population structure without introducing any prior information or assumptions. Population clustering was performed using the STRUCTURE program. Likelihood (ln) increased continuously, with no obvious inflection point (Figure 4A), which implies that the accessions included in the analysis were very diverse. In addition, the Evanno criterion supported the choice of *K* = 2 as the highest level of structure (Figure 4B). There were 273 accessions in subpopulation 1, among which 257 accessions were from China and 16 accessions were from US. By comparison, there were 304 accessions in subpopulation 2, among which 20 accessions were from China and 284 accessions were from the USA (Supplementary Table S3). Of the 20 Chinese accessions in subpopulation 2, the ancestors of 15 of these accessions came from US. These results are consistent with the geographical origin and pedigree information of the 577 accessions.

In order to validate and gain further insight into the genetic diversity of the soybean germplasm panel, we constructed a neighbor-joining tree based on the frequency of shared alleles among the accessions. The 577 soybean accessions were classified into two major groups (Figure 4C). One group was composed largely of accessions from the CN-set, and the other group was mainly composed of accessions from the US-set. These groupings were almost the same as the two subpopulations identified in our analysis conducted with the STRUCTURE program. PCA has been proposed as an alternative to population structure analysis for studying population stratification from genotypic data (Patterson et al., 2006). A PCA of the entire set of 577 accessions with the 5,195 SNPs (Figure 4D and Supplementary Table S4) also showed a clear separation of the same two groups that were identified in our population structure and neighbor-joining tree analyses.

Our unbiased population structure, neighbor-joining tree, and PCA analyses all clearly indicated the existence of two distinct subpopulations among the 577 accessions of our study. Clear divergence existed between the Chinese and US soybean accessions. With the exception of a very small number of accessions that were grouped into the subpopulation for the other country (< 7%), most of the accessions from the same origin (country) were clustered into the same genetic group. Therefore, the following studies were based on the two pre-defined genetic pools (i.e., the original two soybean populations from China and US).

### Genetic differences of the two subpopulations

Genetic differences between the pre-defined genetic pools were evaluated using four types of analysis of population differentiation: AMOVA, Roger genetic distance, genetic relatedness, and allele frequency. AMOVA indicated that 19.33% of the total genetic variation occurred among the subpopulations, whereas 80.67% was within the subpopulations. The population pairwise *F*_ST_ was 0.1933 (*P* < 0.001) between the two subpopulations (Supplementary Table S5), which indicates a high level of difference. The average Roger genetic distances within the CN-set, the US-set, and the combined dataset were 0.3223, 0.2932, and 0.3794, respectively (Figure 4E and Supplementary Table S6). For the CN-set, 42.18% of the distance values were between 0.300 and 0.400. For the US-set 54.99% of the distance values were between 0.200 and 0.300. Viewing the CN-set and the US-set collectively, 67.75% of the distance values were between 0.3 and 0.4 (Figure 4E). These results clearly demonstrate that the genetic distance within the CN-set is larger than that in the US-set, and the genetic distance of pairwise genotypes between subpopulations was larger than that of within subpopulations.

Relative kinship reflects the approximate degree of identity between two given accessions (Figure 4F and Supplementary Table S7). In the CN-set, the kinship coefficients between pairs of accessions varied from 0 to 1.4108, with an overall average of 0.0477; 22.76% of the estimates had a value of zero, which means there is almost no relationship. Comparatively, for the US-set, the kinship coefficients ranged from 0 to 1.5637, with an overall average of 0.0416; 22.43% of the pairwise kinship estimates had a value of zero. For combined analysis of all 577 accessions, the kinship coefficients ranged from 0 to 1.0204, with and overall average of 0.0195; 85.37% of the pairwise kinship estimates had a value of zero, indicating that most of the genotypes were not highly related (Figure 4F).

**Figure 5:**
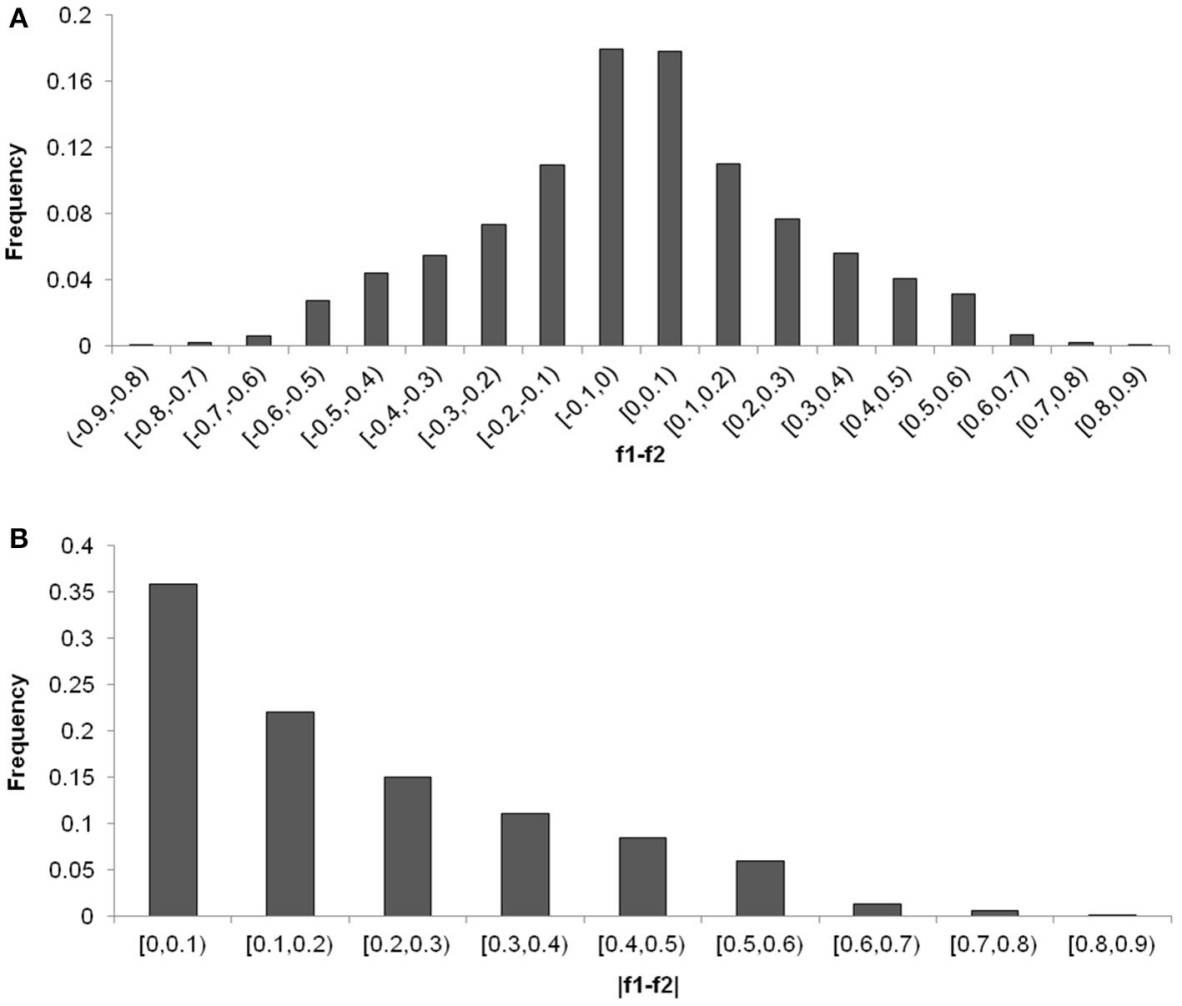
Distribution of the differentiation of allele frequencies, f1-f2 **(A)** and |f1-f2| (**B**, absolute values of f1-f2) between Chinese and American soybean accessions.

**Figure 6:**
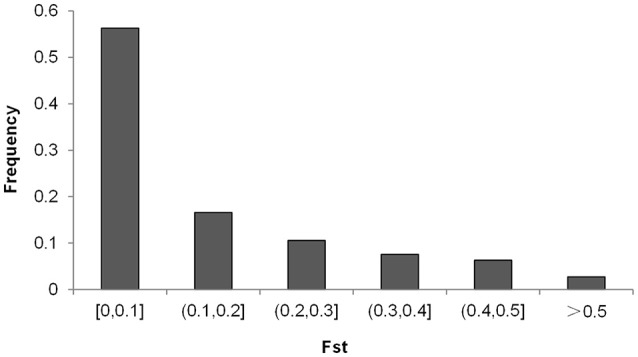
Distribution of pairwise *F*_ST_-values among the two soybean subpopulations.

**Figure 7:**
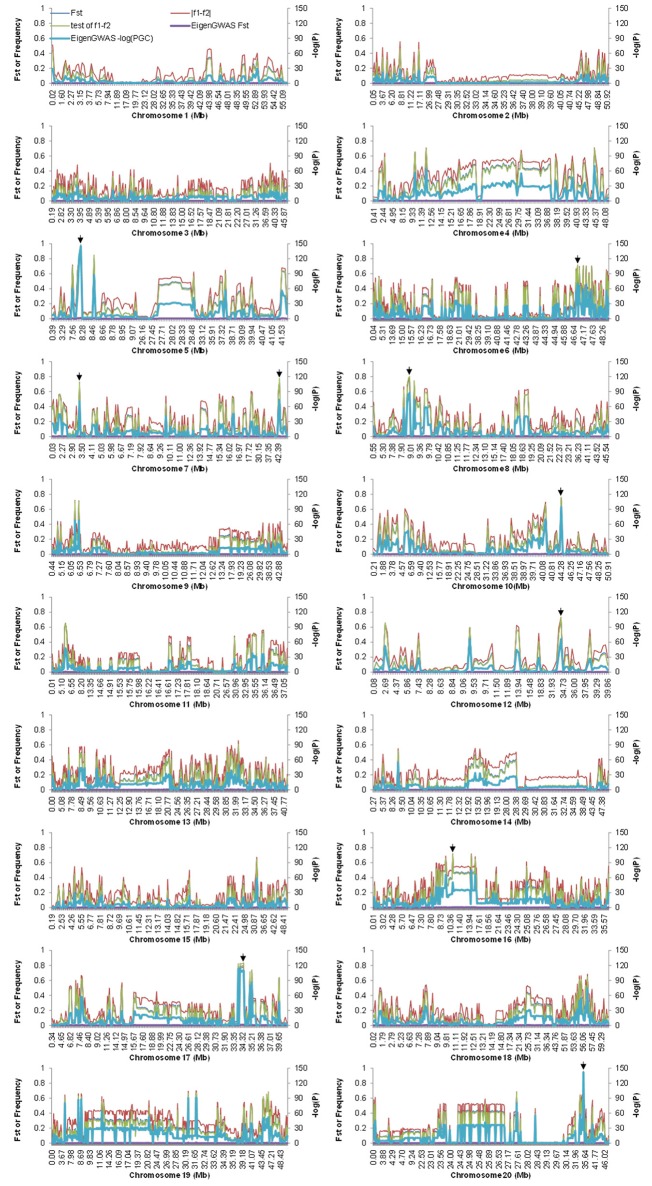
*F*_ST_-values and the allele difference for the whole genome among Chinese and American soybean accessions. *F*_ST_ were calculated from VCFtool and EigenGWAS, denoted by *F*_ST_ and EigenGWAS *F*_ST_, respectively. PGC were *P-*values corrected by genomic control, which were calculated by EigenGWAS. To demonstrate the plot clearly, the values of −log(PGC) were multiplied by 20. Arrows indicate the physical positions of the top 10 candidate loci under selection.

The distribution of the differences of allele frequencies (denoted by *f*_1_−*f*_2_) ranged from 0 to 88.63% between the CN-set and the US-set and was almost symmetrical (Figure 5A), indicating that selection has occurred in both directions. Absolute values of *f*_1_−*f*_2_ for 35.78% of the loci were lower than 0.1, while the values for 7.72% of the loci were higher than 0.5 (Figure 5B). Across the soybean genome, the *F*_ST_ for 56.28% of the loci was lower than 0.1, while the value for 2.73% of the loci was higher than 0.5 (Figure 6). These results imply that a large proportion of genomic regions have maintained similar frequencies in both subpopulations. We performed the statistical tests to determine if the difference between the allele frequencies was significant. Results showed that the allele frequencies of 3,223 SNPs (62.04% of the total 5,195 SNPs) reached the significance level at *P* = 10^−6^. *F*_ST_-values, absolute values of difference between the allele frequencies (|*f*_1_−*f*_2_|), and −log(*P*) of *f*_1_−*f*_2_ values (*P* is the *P*-value from statistical test) are presented in Figure 7. The loci with selection signals identified based on *F*_ST_, test of *f*_1_−*f*_2_ values, and results from EigenGWAS were highly consistent over the 20 soybean chromosomes (Figure 7).

The top 10 candidate loci under selection are presented in Table 2. The strongest selection signal was found at SNP locus ss245627275 on chromosome 5. Four loci with selection signal overlapped with previously identified QTLs for photoperiod traits: ss246094102 on chromosome 6 (Liu et al., 2011), ss246490128 on chromosome 8 (Pooprompan et al., 2006; Reinprecht et al., 2006), ss247294954 on chromosome 10 (Li et al., 2008; Kuroda et al., 2013), and ss250485410 on chromosome 20 (Reinprecht et al., 2006). Seven loci with selection signals overlapped with previously identified QTLs for seed quality. For example, ss250485410 on chromosome 20 is in a region associated with seed protein content (Lu et al., 2013), and seed oil (Qi et al., 2011). Four loci with selection signals overlapped with QTLs associated with defense traits: SNP locus ss246490128 on chromosome 8 is in a region associated with SCN (Wu et al., 2009) and flood tolerance (Sayama et al., 2009); SNP locus ss247294954 is on chromosome 10 and is in a region associated with drought (Carpentieri-Pipolo et al., 2011) and flood tolerance (Githiri et al., 2006); SNP locus ss249030246 on chromosome 16 is in a region associated with whitefly resistance (Zhang et al., 2013); and SNP locus ss250485410 on chromosome 20 is in a regions associated with Sudden Death Syndrome (SDS, Swaminathan et al., 2016). Four loci with selection signals were overlapped with yield-related traits: SNP locus ss246490128 on chromosome 8 is in a region associated with seed weight (Kim et al., 2010), and branching (Liu et al., 2017a); SNP locus ss247294954 on chromosome 10 is in a region associated with seed weight (Sun et al., 2012), and branching (Li et al., 2008); SNP locus ss247790225 on chromosome 12 is in a region associated with branching (Liu et al., 2017a); and SNP locus ss250485410 on chromosome 20 is in a region associated with seed weight (Kato et al., 2014).

**Table 2:** The top 10 SNPs with significantly (*P* < 10^−6^) different allele frequencies between China and the US germplasms.

**SNP name**	**Chr**.	**Allele**	**Position**	**Allele frequency**		**Allele frequency difference**	***F*_ST_**	**Test of *f*_1_−*f*_2_**	**−log(*P*) from EigenGWAS**	**Associated traits**
				**CN**	**US**					
ss245627275^#^	5	A	8234203	0.8967	0.0103	0.8863	0.8866	148.3306	342.8146	
ss246094102	6	A	46849787	0.7955	0.0765	0.7189	0.6925	107.7709	210.3497	Photoperiod insensitivity (Liu et al., 2011); cqSeed protein (Pathan et al., 2013)
ss246165779^*^	7	C	3376487	0.1586	0.8869	0.7283	0.6942	108.3583	209.3325	
ss246411658	7	C	42514762	0.2539	0.9883	0.7344	0.7409	116.1999	221.5186	Seed daidzein (Gutierrez-Gonzalez et al., 2010)
ss246490128	8	C	8917276	0.8971	0.1160	0.7810	0.7567	120.5933	269.0964	Pod maturity (Reinprecht et al., 2006); First flower (Pooprompan et al., 2006); Seed weight (Kim et al., 2010); Branching (Liu et al., 2017a); Seed coat and hilum color (Zhou et al., 2015); Seed protein (Lu et al., 2013); SCN (Wu et al., 2009); Flood tolerance (Sayama et al., 2009)
ss247294954	10	C	44278194	0.0879	0.8426	0.7546	0.7256	114.3218	296.2328	First flower (Kuroda et al., 2013); Reproductive stage length (Li et al., 2008); Pod maturity (Li et al., 2008); Seed weight (Sun et al., 2012); Branching (Li et al., 2008); Seed protein (Chen et al., 2007); Drought tolerance (Carpentieri-Pipolo et al., 2011); Flood tolerance (Githiri et al., 2006)
ss247790225	12	A	34375177	0.1350	0.8672	0.7322	0.6971	108.8819	196.7905	Branching (Liu et al., 2017a); Seed oil (Liu et al., 2017a); Seed protein (Liu et al., 2017a); Lodging (Liu et al., 2017b)
ss249030246	16	A	10794262	0.9233	0.2040	0.7193	0.6777	106.5537	142.4584	Seed oil (Mao et al., 2013); Whitefly resistance (Zhang et al., 2013)
ss249429323^※^	17	A	34433642	0.1097	0.9078	0.7982	0.7783	124.9834	301.3143	
ss250485410	20	C	34577475	0.0967	0.9680	0.8713	0.8671	143.8360	294.0404	First flower (Reinprecht et al., 2006); Seed weight (Kato et al., 2014); Seed protein (Lu et al., 2013); Seed oil (Qi et al., 2011); Lodging (Liu et al., 2017b); Sudden Death Syndrome (SDS, Swaminathan et al., 2016)

# Gene annotation information for this locus is “RAS-related nuclear protein,” and “Cytochrome P450 superfamily protein”;

* Gene annotation information for this locus is “nucleoside diphosphate kinase,” “Small nuclear ribonucleoprotein splicing factor,” and “Lung seven transmembrane receptor”;

※ *Gene annotation information for this locus is “Ubiquinol cytochrome c reductase, subunit QCR7,” and “protein domain specific binding.” CN is for China; and US is for United States of America*.

Three loci with selection signals (ss246490128 on chromosome 8, ss247294954 on chromosome 10, and ss250485410 on chromosome 20) had pleiotropic effects on all of the following physiological aspects: photoperiod, seed quality, defense, and yield-related traits. Among the top 10 loci ranked based on the strength of their selection signals, ss245627275 on chromosome 5, ss246165779 on chromosome 7, and ss249429323 on chromosome 17 have not been previously reported to be associated with any traits, and one gene near SNP locus ss245627275 was annotated as RAS-related nuclear protein; one gene near SNP locus ss246165779 was annotated as having a function relating to nucleoside diphosphate kinase activity; and one gene near SNP locus ss249429323 was annotated as having a function relating to protein domain specific binding (Table 2). The calculation of the allele frequency for the two subpopulations allowed the identification of subpopulation-specific alleles. Of the 5,191 SNP markers, four specific alleles for the CN-set were distributed on chromosomes 5, 7, 10, and 12, but no specific allele existed in the US-set (Table 3).

**Table 3:** Population-specific alleles for Chinese soybean genotypes.

**Marker**	**Allele**	**Chr**.	**Position**	**Allele frequency in CN**	**Associated trait**
ss245630590	C	5	8603833	0.0939	
ss246176034	G	7	4488650	0.0421	First flower (Orf et al., 1999); Pod maturity (Wang et al., 2004); Seed yield (Wang et al., 2014)
ss247314990	G	10	47212732	0.0722	Pod maturity (Specht et al., 2001); Seed weight (Han et al., 2012)
ss247667029	G	12	8693064	0.0618	Seed isoflavone (Han et al., 2015)

*CN is for China*.

## Discussion

Fang et al. (2017) dissected the genetic architecture of 84 agronomic traits and investigated the genetic networks underling their phenotypic correlations by 809 soybean accessions with diverse geographic distribution. Of 809 accessions, only 67 accessions derived from US, and majority of accessions (683 accessions) came from China, accounting for 84.4%. While in current study, to particularly compare the genetic diversity of soybean cultivars from China and US, and to identify loci that have undergone selection based on different statistical algorithms, we collected fairly balanced samples (277 for China vs. 300 for US; Figure 1) from the major production areas in both countries to reach a high statistical power. The planting area of soybean in northeast and Huang-huai-hai regions is account for more than 80% of total soybean planting area in China. Of 1,300 cultivars released in China from 2005 to 2013, 1,077 cultivars (82.85%) were from northeast and Huang-huai-hai regions, and 656 cultivars (50.46%) were the progenies of cultivars imported from abroad. While for the southern region, 223 cultivars were released accounting for 17.15% of the total cultivars released in China, of which 82 cultivars (6.31%) were released with exotic ancestors. As for the materials collected in USA, the situation is similar as those in China. The main production areas in USA are in the central and northern regions, and the released cultivars from southern region of the USA are comparatively small. Considering the reasons above, the 277 accessions in the CN-set were selected from the geographic regions typified by the Northern spring (Nsp) ecotype and the Huang-huai-hai summer (Hsu) ecotype. The 300 accessions in the US-set covered accessions in maturity groups I–IV, which include accessions used in the main soybean production regions of the USA. It is important to consider the strong influence of maturity on phenotypic traits in soybean: large pleiotropic effects are well-documented for plant height, lodging, and seed yield, and these large effects tend to overwhelm other phenotypic differences among genotypes (Cui et al., 2001). However, importantly, identification based on genomic data is not influenced by maturity or other phenotypic factors. In the present study, 5,361 SNP markers from the Illumina SoySNP6k iSelect BeadChip were employed to genotype all of the accessions in our diversity panel.

### Genetic diversity among soybean groups

The average genetic diversity and PIC-values for the combined set of all accessions were, respectively, 0.3489 and 0.2769. Compared to the previously reported results based on SNP (Hao et al., 2012; Zhou et al., 2015) or SSR (Li et al., 2011) data, the level of genetic diversity that we observed here is lower, almost certainly because the materials used in the present study were cultivars and advanced breeding lines, while the materials used in Hao et al. (2012) and Li et al. (2011) were mainly landraces. We observed that the accessions from the US had lower genetic diversity and PIC-values than the accessions from China. Our conclusion that the Chinese cultivars are more diverse than the American cultivars is consistent with the conclusions of previous studies, including molecular analyses using RAPD markers to predict the likely ancestors of Chinese and US soybean cultivars (Qiu et al., 1997; Li et al., 2001) and a phenotypic diversity study of modern Chinese and North American soybean cultivars conducted by Cui et al. (2001). The lower LD-values and larger genetic distance within the Chinese accessions as compared to the American accessions again highlights that the Chinese germplasm has relatively greater genetic diversity.

### Population structure analysis of the chinese and US soybean accessions

AMOVA, model-based population structure analysis, NJ-cluster analysis, and PCA were used to examine whether or not the 577 soybean accessions were from highly diverse origins and/or whether or not the accessions from China and the USA are homogeneous or represent two genetically distinct subgroups. We used a variety of methods and consistently obtained similar results for both the number of accessions and the particular membership of accessions within groups. The American accessions were genetically distinct from the Chinese accessions. There were clearly two different subpopulations: an American one and a Chinese one. Cluster analyses grouped accessions with similar geographical origins together, and these findings were in accordance with previous studies (Li et al., 2011; Hao et al., 2012; Sun et al., 2014; Zhou et al., 2015; Fang et al., 2017; Liu et al., 2017a). We noted that 93.76% of the soybean accessions were assigned into the subgroup corresponding to their expected geographical origin (Supplementary Tables S1–S3), implying that the geographic distribution of soybean cultivars and advanced breeding lines is a reliable parameter to use for understanding the overall population structure.

Regarding kinship, the 577 accessions are distantly related: For combined analysis of the two subpopulations, 85.37% of the pairwise kinship estimates were equal to zero (Figure 4F). The distance between the two subpopulations (0.3794; Supplementary Table S6) was larger than that within either of the two individual groupings (0.3223 for the CN-set and 0.2932 for the US-set; Supplementary Table S6). Our conclusion that the accessions are distantly related is also supported by the significant allelic frequency differences observed among the accessions based on AMOVA and *F*_ST_ analyses. Significant structuring of genetic variation was found between the Chinese and American soybean accessions (Supplementary Table S5). Viewed collectively, our findings indicate that even though the soybean genetic base in the USA is ultimately derived from only a few soybean cultivars that were introduced from China (Li et al., 2001), and Chinese plant breeders have successfully employed elite Northern American cultivars as breeding materials (Gai et al., 2015), breeding efforts of breeders of two countries in the time have generated a quite distinct genetic base of soybeans. Another influence that may underlie the observed genetic diversity between the two subpopulations is the efforts of breeders over many decades that have been focused on improving local adaptation to different environmental conditions in the soybean growth areas of the two countries. Given the recognized importance of including materials from diverse genetic groups in breeding programs (Thompson and Nelson, 1998; Li et al., 2001), it is important to carefully support the exchange, identification, and utilization of exotic soybean germplasm in soybean improvement efforts.

### Implications for soybean breeding

The identification of loci with selective signals is a centrally important step for understanding how various populations have adapted to particular agronomic practices and/or unique environments. The *F*_ST_ approach for detecting loci that have been under selection has been applied to many crops, including wild emmer wheat (Ren et al., 2013), common bean (Papa et al., 2007), tomato (Corrado et al., 2013), and soybean (Han et al., 2016), among many others, and markers that are identified using an *F*_ST_-outlier method often reveal genome regions that have previously been associated with quantitative trait loci (QTL) related to domestication (Ren et al., 2013).

In the present study, we used an *F*_ST_-based statistical analysis and EigenGWAS to identify selection signals at SNP loci. Although, the majority of the loci had low *F*_ST_-values in pairwise comparisons, our data revealed the presence of genomic regions with high genetic variation between the two subpopulations (Figure 6), and the results were consistent with that of EigenGWAS. Of the top 10 candidate loci under selection, four loci overlapped with previously identified QTLs for photoperiod traits, seven loci overlapped with previously identified QTLs for seed quality-related traits, four loci were in regions associated with abiotic and biotic stress related traits, and four loci were in regions associated with yield traits. Three of the loci with selection signals were in regions associated with multiple physiological aspects (photoperiod, seed quality, abiotic and biotic-related, and yield-related traits). One locus ss246490128 on chromosome 8 overlapped with previously identified QTL Sat_215, around which a candidate domestication gene *Glymo08g09310* controlled seed size was identified (Zhou et al., 2016). Zhou et al. (2015) also reported four loci related to soybean domestication, which were in the regions of loci ss246490128 on Chromosome 8, ss247294954 on Chromosome 10, ss247790225 on chromosome 12, and ss249429323 on chromosome 17 in present study, respectively. The trait-related loci under selection can result in phenotypic variation between populations from two countries. For example, Wang et al. (2005) evaluated the yield-related traits for accessions from China and the USA that were from similar latitudes, and found that cultivars from the USA had higher plant heights, more branching, higher numbers of pods, and higher yields than those from China. The loci with selection signals that we observed may be (or have been) under direct selection, but it is more likely that they are located in chromosome regions that were selected during the domestication process. The putative functions associated with these loci are that related to the role in adaptation: these loci are putatively involved in processes that are vital for plant growth and survival. The loci with selection signals that we identified in the present study are of potential interest for plant breeders, as they likely contribute to the existing crop performance differences between Chinese and US soybeans.

## Author contributions

LQ, DW, ZL, and SW: conceived and designed the experiments; ZL, ZW, and XF: performed the experiments; ZL, HL, YL, RG, and YG: analyzed the data; ZL, HL, and ZW: wrote the paper.

### Conflict of interest statement

The authors declare that the research was conducted in the absence of any commercial or financial relationships that could be construed as a potential conflict of interest.
