# Supplementary material for: Comparison of Genetic Diversity between Chinese and American Soybean (Glycine max (L.)) Accessions Revealed by High-Density SNPs
Source: Front Plant Sci. 2017 Nov 30;8:2014. doi: 10.3389/fpls.2017.02014 (PMC5715234; doi:10.3389/fpls.2017.02014)
Supplement: Supplementary file 1 [file Table1.DOCX]

Supplementary Table S1 Detailed information for the 277 soybean accessions from China.

| Test ID | Name | Geographic  source | Released year |
| --- | --- | --- | --- |
| C001 | Sinong 2 | Jilin | 2001 |
| C002 | Jiyu 72 | Jilin | 2004 |
| C003 | Jiyu 74 | Jilin | 2005 |
| C004 | Jilin 36 | Jilin | 1996 |
| C005 | Jikedou 7 | Jilin | 2004 |
| C006 | Haodou 2000 | Jilin | 2005 |
| C007 | Jifeng 2 | Jilin | 2000 |
| C008 | Zhonghuang 24 | Beijing | 2003 |
| C009 | Jinong 13 | Jilin | 2003 |
| C010 | Jilin 38 | Jilin | 1998 |
| C011 | Jinong 14 | Jilin | 2003 |
| C012 | Liaodou 4 | Liaoning | 1989 |
| C013 | Jiunong 23 | Jilin | 2000 |
| C014 | Jilin 45 | Jilin | 2000 |
| C015 | Jinong 7 | Jilin | 1999 |
| C016 | Jinong 11 | Jilin | 2002 |
| C017 | Ji 1005 | Jilin | 2001 |
| C018 | Jiyu 70 | Jilin | 2003 |
| C019 | Jiunong 31 | Jilin | 2005 |
| C020 | Jiunong 29 | Jilin | 2003 |
| C021 | Fu 97-16 | Liaoning | 2002 |
| C022 | Jiunong 22 | Jilin | 1999 |
| C023 | Jiunong 33 | Jilin | 2005 |
| C024 | Tongnong 7 | Jilin | 1978 |
| C025 | Jiunong 24 | Jilin | 2001 |
| C026 | Jilin 39 | Jilin | 1998 |
| C027 | Jinong 15 | Jilin | 2004 |
| C028 | Jinong 8 | Jilin | 2000 |
| C029 | Jilin 35 | Jilin | 1995 |
| C030 | Jiyu 52 | Jilin | 2001 |
| C031 | Changnong 8 | Jilin | 1996 |
| C032 | Changnong 11 | Jilin | 2000 |
| C033 | Jiunong 27 | Jilin | 2002 |
| C034 | Tongnong 5 | Jilin | 1978 |
| C035 | Tongnong 14 | Jilin | 2001 |
| C036 | Changnong 15 | Jilin | 2002 |
| C037 | Tongnong 13 | Jilin | 2001 |
| C038 | Tongnong 6 | Jilin | 1978 |
| C039 | Jiunong 25 | Jilin | 2002 |
| C040 | Jiunong 21 | Jilin | 1995 |
| C041 | Tongnong 11 | Jilin | 1995 |
| C042 | Jilin 34 | Jilin | 1996 |
| C043 | Tongnong 12 | Jilin | 2000 |
| C044 | Jiyu 50 | Jilin | 2001 |
| C045 | Changnong 10 | Jilin | 2000 |
| C046 | Jiunong 28 | Jilin | 2003 |
| C047 | Jiyu 71 | Jilin | 2003 |
| C048 | Jiyu 54 | Jilin | 2001 |
| C049 | Jiyu 67 | Jilin | 2002 |
| C050 | Jiyu 57 | Jilin | 2001 |
| C051 | Jiyu 58 | Jilin | 2001 |
| C052 | Jilinxiaoli 6 | Jilin | 2002 |
| C053 | Yannong 10 | Jilin | 2002 |
| C054 | Jilin 49 | Jilin | 2000 |
| C055 | Yannong 11 | Jilin | 2003 |
| C056 | Bainong 10 | Jilin | 2004 |
| C057 | Jilin 47 | Jilin | 1999 |
| C058 | Jiyu 64 | Jilin | 2002 |
| C059 | Jilin 32 | Jilin | 1995 |
| C060 | Changnong 19 | Jilin | 2005 |
| C061 | Jiyu 59 | Jilin | 2001 |
| C062 | Jifeng 4 | Jilin | 2005 |
| C063 | Jilin 46 | Jilin | 1999 |
| C064 | Jiyu 68 | Jilin | 2003 |
| C065 | Jikedou 5 | Jilin | 2003 |
| C066 | Jiyu 66 | Jilin | 2002 |
| C067 | Jiyu 63 | Jilin | 2002 |
| C068 | Jilinxiaoli 7 | Jilin | 2004 |
| C069 | Bainong 6 | Jilin | 1994 |
| C070 | Bainong 8 | Jilin | 1998 |
| C071 | Jilin 43 | Jilin | 1998 |
| C072 | Bainong 9 | Jilin | 1999 |
| C073 | Jikedou 3 | Jilin | 2002 |
| C074 | Yannong 8 | Jilin | 1999 |
| C075 | Bainong 7 | Jilin | 1996 |
| C076 | Jiyu 80 | Jilin | 2005 |
| C077 | Jiyu 86 | Jilin | 2009 |
| C078 | Jiyu 401 | Jilin | 2010 |
| C079 | Jiyu 89 | Jilin | 2007 |
| C080 | Jiyu 93 | Jilin | 2008 |
| C081 | Jiyu 75 | Jilin | 2005 |
| C082 | Jiyu 301 | Jilin | 2009 |
| C083 | Jiyu 201 | Jilin | 2011 |
| C084 | Jiyu 83 | Jilin | 2006 |
| C085 | Jiyu 84 | Jilin | 2006 |
| C086 | Jiyu 79 | Jilin | 2005 |
| C087 | Kangxian 5 | Heilongjiang | 2003 |
| C088 | Kenjiandou 1 | Heilongjiang | 1987 |
| C089 | Huajiang 1 | Heilongjiang | 2005 |
| C090 | Kenfeng 10 | Heilongjiang | 2003 |
| C091 | Kenjian 27 | Heilongjiang | 2003 |
| C092 | Kenfeng 13 | Heilongjiang | 2005 |
| C093 | Heinong 43 | Heilongjiang | 2002 |
| C094 | Kennong 11 | Heilongjiang | 2003 |
| C095 | Heinong 41 | Heilongjiang | 1999 |
| C096 | Heisheng 101 | Heilongjiang | 1997 |
| C097 | Suinong 16 | Heilongjiang | 2000 |
| C098 | Kenjiandou 15 | Heilongjiang | 2000 |
| C099 | Heihe 19 | Heilongjiang | 1998 |
| C100 | Heinong 44 | Heilongjiang | 2002 |
| C101 | Hefeng 40 | Heilongjiang | 2000 |
| C102 | Kenjiandou 25 | Heilongjiang | 2003 |
| C103 | Dongda 1 | Heilongjiang | 2003 |
| C104 | Hefeng 43 | Heilongjiang | 2002 |
| C105 | Kenfeng 7 | Heilongjiang | 2001 |
| C106 | Heihe 26 | Heilongjiang | 2001 |
| C107 | Hefeng 25 | Heilongjiang | 1984 |
| C108 | Suinong 14 | Heilongjiang | 1996 |
| C109 | Suinong 21 | Heilongjiang | 2004 |
| C110 | Kenfeng 6 | Heilongjiang | 2001 |
| C111 | Hongfeng 8 | Heilongjiang | 1993 |
| C112 | Suinong 17 | Heilongjiang | 2001 |
| C113 | Heihe 27 | Heilongjiang | 2002 |
| C114 | Kenjiandou 23 | Heilongjiang | 2002 |
| C115 | Heihe 14 | Heilongjiang | 1996 |
| C116 | Heihe 31 | Heilongjiang | 2003 |
| C117 | Suinong 18 | Heilongjiang | 2002 |
| C118 | Heihe 32 | Heilongjiang | 2004 |
| C119 | Hefeng 42 | Heilongjiang | 2002 |
| C120 | Heihe 35 | Heilongjiang | 2004 |
| C121 | Heihe 30 | Heilongjiang | 2003 |
| C122 | Dongnong 42 | Heilongjiang | 2000 |
| C123 | Kenjiandou 14 | Heilongjiang | 2000 |
| C124 | Beifeng 14 | Heilongjiang | 1997 |
| C125 | Beifeng 13 | Heilongjiang | 1996 |
| C126 | Jiufeng 9 | Heilongjiang | 2003 |
| C127 | Beifeng 9 | Heilongjiang | 1995 |
| C128 | Heinong 38 | Heilongjiang | 1992 |
| C129 | Beidou 30 | Heilongjiang | 2008 |
| C130 | Heihe 38 | Heilongjiang | 2005 |
| C131 | Beidou 38 | Heilongjiang | 2011 |
| C132 | Heihe 25 | Heilongjiang | 2001 |
| C133 | Beidou 35 | Heilongjiang | 2009 |
| C134 | Beidou 1 | Heilongjiang | 2005 |
| C135 | Beidou 18 | Heilongjiang | 2008 |
| C136 | Suinong 19 | Heilongjiang | 2002 |
| C137 | Beifeng 11 | Heilongjiang | 1995 |
| C138 | Jikedou 1 | Jilin | 2001 |
| C139 | Hongfeng 7 | Heilongjiang | 1993 |
| C140 | Kenjiandou 22 | Heilongjiang | 2002 |
| C141 | Beifeng 17 | Heilongjiang | 2004 |
| C142 | Heihe 17 | Heilongjiang | 2000 |
| C143 | Changnong 9 | Jilin | 1998 |
| C144 | Jiunong 20 | Jilin | 1995 |
| C145 | Hefeng 36 | Heilongjiang | 1996 |
| C146 | Kenjiandou 31 | Heilongjiang | 2004 |
| C147 | Longxiaoli 1 | Heilongjiang | 2003 |
| C148 | Jiufeng 8 | Heilongjiang | 1998 |
| C149 | Heyin 1 | Anhui |  |
| C150 | Heyin 2 | Anhui |  |
| C151 | Yudou 12 | Henan | 1992 |
| C152 | Ludou 8 | Shandong | 1988 |
| C153 | Tiegan 1 | Shandong |  |
| C154 | Wenfeng 1 | Shandong |  |
| C155 | Yudou 1 | Henan | 1985 |
| C156 | Zaoshu 17 | Beijing | 1989 |
| C157 | Zhonghuang 3 | Beijing | 1990 |
| C158 | Zhongpin 661 | Beijing | 1994 |
| C159 | Jindou 22 | Shanxi | 1998 |
| C160 | Jindou 26 | Shanxi | 2001 |
| C161 | Jindou 28 | Shanxi | 2004 |
| C162 | Jinyi 30 | Shanxi | 2004 |
| C163 | Jinda 74 | Shanxi | 2004 |
| C164 | Kexin 5 | Beijing | 2000 |
| C165 | Liaodou 14 | Liaoning | 2003 |
| C166 | Tiefeng 31 | Liaoning | 2001 |
| C167 | Dongdou 1 | Liaoning | 2005 |
| C168 | Liaoshou 2 | Liaoning | 2005 |
| C169 | Tiefeng 28 | Liaoning | 1996 |
| C170 | Zhonghuang 13 | Beijing | 2001 |
| C171 | Zhonghuang 19 | Beijing | 2003 |
| C172 | Zhonghuang 20 | Beijing | 2001 |
| C173 | Zhongpin 662 | Beijing | 2002 |
| C174 | Jidou 12 | Hebei | 1996 |
| C175 | Wuxing 1 | Hebei | 2001 |
| C176 | Wuxing 2 | Hebei | 2004 |
| C177 | Handou 3 | Hebei | 1999 |
| C178 | Handou 5 | Hebei | 2004 |
| C179 | Huayou 542 | Hebei | 1999 |
| C180 | Jindou 29 | Shanxi | 2004 |
| C181 | Ludou 10 | Shandong | 1993 |
| C182 | Ludou 11 | Shandong | 1995 |
| C183 | Qichadou 2 | Shandong | 2002 |
| C184 | Hedou 13 | Shandong | 2005 |
| C185 | Gaofeng 1 | Shandong | 2005 |
| C186 | 84-51 | Shandong | 1995 |
| C187 | Yudou 15 | Henan | 1993 |
| C188 | Yudou 19 | Henan | 1995 |
| C189 | Yudou 20 | Henan | 1995 |
| C190 | Zheng 90007 | Henan | 2001 |
| C191 | Xudou 8 | Jiangsu | 1996 |
| C192 | Xudou 11 | Jiangsu | 2002 |
| C193 | Nannong 217 | Jiangsu | 1996 |
| C194 | Jinda 70 | Shanxi | 2003 |
| C195 | Changnong 17 | Jilin | 1998 |
| C196 | Jilin 20 | Jilin | 1985 |
| C197 | Jilin 33 | Jilin | 1995 |
| C198 | Jihuang 60 | Jilin |  |
| C199 | Jilinxiaoli 4 | Jilin | 2000 |
| C200 | Jiyuanyin 3 | Jilin | 1999 |
| C201 | Heinong 30 | Heilongjiang | 1987 |
| C202 | Heinong 35 | Heilongjiang | 1990 |
| C203 | Heinong 46 | Heilongjiang | 2003 |
| C204 | Heinong 48 | Heilongjiang | 2004 |
| C205 | Dongnong 34 | Heilongjiang | 1982 |
| C206 | Dongnong 43 | Heilongjiang | 1999 |
| C207 | Dongnong 44 | Heilongjiang | 2000 |
| C208 | Dongnong 46 | Heilongjiang | 2003 |
| C209 | Dongsheng 1 | Heilongjiang | 2003 |
| C210 | Suinong 4 | Heilongjiang | 1981 |
| C211 | Suinong 8 | Heilongjiang | 1989 |
| C212 | Suinong 10 | Heilongjiang | 1994 |
| C213 | Suinong 11 | Heilongjiang | 1995 |
| C214 | Suinong 15 | Heilongjiang | 1998 |
| C215 | Kennong 7 | Heilongjiang | 1994 |
| C216 | Kennong 16 | Heilongjiang | 1998 |
| C217 | Kennong 17 | Heilongjiang | 2001 |
| C218 | Kennong 18 | Heilongjiang | 2001 |
| C219 | Kennong 19 | Heilongjiang | 2002 |
| C220 | Kenjiandou 4 | Heilongjiang | 1999 |
| C221 | Kenjiandou 26 | Heilongjiang | 2003 |
| C222 | Kenjiandou 27 | Heilongjiang | 2003 |
| C223 | Kenjiandou 33 | Heilongjiang | 2004 |
| C224 | Kenfeng 9 | Heilongjiang | 2002 |
| C225 | Kenfeng 11 | Heilongjiang | 2003 |
| C226 | Hongfeng 11 | Heilongjiang | 1998 |
| C227 | Hongfeng 12 | Heilongjiang | 2003 |
| C228 | Hefeng 27 | Heilongjiang | 1986 |
| C229 | Hefeng 30 | Heilongjiang | 1988 |
| C230 | Hefeng 35 | Heilongjiang | 1994 |
| C231 | Hefeng 39 | Heilongjiang | 2000 |
| C232 | Hefeng 44 | Heilongjiang | 2003 |
| C233 | Hefeng 45 | Heilongjiang | 2003 |
| C234 | Nenfeng 10 | Heilongjiang | 1981 |
| C235 | Nenfeng 13 | Heilongjiang | 1987 |
| C236 | Nenfeng 17 | Heilongjiang | 2004 |
| C237 | Nenliang 7 | Heilongjiang |  |
| C238 | Beifeng 2 | Heilongjiang | 1983 |
| C239 | Beifeng 16 | Heilongjiang | 2002 |
| C240 | Baofeng 7 | Heilongjiang | 1994 |
| C241 | Baofeng 8 | Heilongjiang | 1995 |
| C242 | Fengshou 1 | Heilongjiang | 1958 |
| C243 | Fengshou 8 | Heilongjiang | 1996 |
| C244 | Fengshou 9 | Heilongjiang | 1996 |
| C245 | Fengshou 10 | Heilongjiang | 1966 |
| C246 | Fengshou 12 | Heilongjiang | 1969 |
| C247 | Fengshou 14 | Heilongjiang | 1970 |
| C248 | Fengshou 18 | Heilongjiang | 1981 |
| C249 | Fengshou 19 | Heilongjiang | 1985 |
| C250 | Jiufeng 1 | Heilongjiang | 1983 |
| C251 | Jiufeng 3 | Heilongjiang | 1986 |
| C252 | Jiufeng 4 | Heilongjiang | 1988 |
| C253 | Jiufeng 6 | Heilongjiang | 1995 |
| C254 | Jiufeng 7 | Heilongjiang | 1996 |
| C255 | Heihe 3 | Heilongjiang | 1966 |
| C256 | Heihe 4 | Heilongjiang | 1982 |
| C257 | Heihe 5 | Heilongjiang | 1986 |
| C258 | Heihe 7 | Heilongjiang | 1988 |
| C259 | Heihe 11 | Heilongjiang | 1994 |
| C260 | Heihe 18 | Heilongjiang | 1998 |
| C261 | Heihe 23 | Heilongjiang | 2000 |
| C262 | Heihe 28 | Heilongjiang | 2003 |
| C263 | Heihe 29 | Heilongjiang | 2003 |
| C264 | Heihe 34 | Heilongjiang | 2004 |
| C265 | Heihe 54 | Heilongjiang | 1967 |
| C266 | Neidou 4 | Neimeng | 1994 |
| C267 | Mengdou 11 | Neimeng | 2002 |
| C268 | Mengdou 13 | Neimeng | 2003 |
| C269 | Mengdou 14 | Neimeng | 2004 |
| C270 | Beihudou | Heilongjiang | 1972 |
| C271 | Kaiyu 12 | Liaoning | 2000 |
| C272 | Tiefeng 33 | Liaoning | 2003 |
| C273 | Zhonghuang 28 | Beijing | 2004 |
| C274 | Kefeng 14 | Beijing | 2001 |
| C275 | Zhonghuang 57 | Beijing | 2010 |
| C276 | Zhonghuang 35 | Beijing | 2006 |
| C277 | Tiefeng 8 | Liaoning | 1970 |
