# Supplementary material for: Comparison of Genetic Diversity between Chinese and American Soybean (Glycine max (L.)) Accessions Revealed by High-Density SNPs
Source: Front Plant Sci. 2017 Nov 30;8:2014. doi: 10.3389/fpls.2017.02014 (PMC5715234; doi:10.3389/fpls.2017.02014)
Supplement: Supplementary file 2 [file Table2.DOCX]

Supplementary Table S2 Detailed information for the 300 soybean accessions from the United States.

| Test ID | Varietyname | Source | Maturity Group |
| --- | --- | --- | --- |
| A001 | AR10SDS | Iowa State University | I |
| A002 | M00-456052-1 | Minnesota Agricultural Experiment Station | I |
| A003 | M97-357138 | Minnesota Agricultural Experiment Station | I |
| A004 | MN1606SP | Minnesota Agricultural Experiment Station | I |
| A005 | Venus RR | MERSCHMAN | I |
| A006 | Myc5171RR-1 | Resistance check | I |
| A007 | AR07-176037 | Iowa State University | I |
| A008 | AR09-292004-1 | Iowa State University | I |
| A009 | M04-342068 | Minnesota Agricultural Experiment Station | I |
| A010 | M03-326084 | Minnesota Agricultural Experiment Station | I |
| A011 | Hodgson | The Ohio State University | I |
| A012 | M04-287017 | Minnesota Agricultural Experiment Station | I |
| A013 | M04-295008 | Minnesota Agricultural Experiment Station | I |
| A014 | M04-372-14004 | Minnesota Agricultural Experiment Station | I |
| A015 | M03-347183 | Minnesota Agricultural Experiment Station | I |
| A016 | M03-364021 | Minnesota Agricultural Experiment Station | I |
| A017 | AR03-263051 | Iowa State University | II |
| A018 | 233+ RR | Resistance check | II |
| A019 | 2900CR-1 | Susceptible check | II |
| A020 | H-2494 | Susceptible check | II |
| A021 | LD03-23508R | University of Illinois | II |
| A022 | LD06-50113R-1 | University of Illinois | II |
| A023 | E07080 | Michigan State University | II |
| A024 | E07040 | Michigan State University | II |
| A025 | GD2422 | Michigan State University | II |
| A026 | GD0172 | Michigan State University | II |
| A027 | GD1943 | Michigan State University | II |
| A028 | E05144 | Michigan State University | II |
| A029 | E05226-T | Michigan State University | II |
| A030 | E06246 | Michigan State University | II |
| A031 | E09014 | Michigan State University | II |
| A032 | E09017 | Michigan State University | II |
| A033 | LD08-12419a | University of Illinois | II |
| A034 | LD08-12428a | University of Illinois | II |
| A035 | LD08-12430a | University of Illinois | II |
| A036 | LD08-12435a | University of Illinois | II |
| A037 | LD08-12438a | University of Illinois | II |
| A038 | LD08-12441a | University of Illinois | II |
| A039 | LD08-12442a | University of Illinois | II |
| A040 | LD08-12445a | University of Illinois | II |
| A041 | LD08-12446a | University of Illinois | II |
| A042 | LD08-3243 | University of Illinois | II |
| A043 | LD08-3994 | University of Illinois | II |
| A044 | LD08-6982 | University of Illinois | II |
| A045 | LD08-2370 | University of Illinois | II |
| A046 | LD08-2388 | University of Illinois | II |
| A047 | LD08-4202 | University of Illinois | II |
| A048 | AR08-285024 | Iowa State University | II |
| A049 | AR07-276022 | Iowa State University | II |
| A050 | AR10-206115 | Iowa State University | II |
| A051 | AR10-206121 | Iowa State University | II |
| A052 | AR10-206128 | Iowa State University | II |
| A053 | AR03-361091-1 | Iowa State University | III |
| A054 | AR07-376031-1 | Iowa State University | III |
| A055 | Morgan | Susceptible check | III |
| A056 | LS05-0220 | Southern Illinois University Carbondale | III |
| A057 | 2900CR-2 | Susceptible check | III |
| A058 | LD06-14178R | University of Illinois | III |
| A059 | LD08-12425a | University of Illinois | III |
| A060 | LD08-12426a | University of Illinois | III |
| A061 | LD08-1566 | University of Illinois | III |
| A062 | LD08-1592 | University of Illinois | III |
| A063 | LD08-1646 | University of Illinois | III |
| A064 | LD08-1673 | University of Illinois | III |
| A065 | LD08-2355 | University of Illinois | III |
| A066 | LD08-3611 | University of Illinois | III |
| A067 | LD08-3936 | University of Illinois | III |
| A068 | LD08-4767a | University of Illinois | III |
| A069 | LD08-5579 | University of Illinois | III |
| A070 | LD08-6068a | University of Illinois | III |
| A071 | LD08-6972 | University of Illinois | III |
| A072 | LD08-8622 | University of Illinois | III |
| A073 | LD08-756 | University of Illinois | III |
| A074 | LD08-871 | University of Illinois | III |
| A075 | LD07-2014 | University of Illinois | III |
| A076 | LS08-3430 | Southern Illinois University Carbondale | III |
| A077 | LS08-3817 | Southern Illinois University Carbondale | III |
| A078 | LS08-3849 | Southern Illinois University Carbondale | III |
| A079 | LS08-5828 | Southern Illinois University Carbondale | III |
| A080 | SS05-5228 | University of Missouri | III |
| A081 | SS06-7843 | University of Missouri | III |
| A082 | SS07-18100 | University of Missouri | III |
| A083 | SS07-15994 | University of Missouri | III |
| A084 | SS08-2570 | University of Missouri | III |
| A085 | SS08-3279 | University of Missouri | III |
| A086 | SS08-2558 | University of Missouri | III |
| A087 | AR09-392007 | Iowa State University | III |
| A088 | AR10-306029 | Iowa State University | III |
| A089 | Ripley | USDA-ARS | IV |
| A090 | LS94-3207 | Southern Illinois University Carbondale | IV |
| A091 | Spencer | Susceptible check | IV |
| A092 | CM497 | Iowa State University | IV |
| A093 | LD06-7609 | University of Illinois | IV |
| A094 | LD06-7620 | University of Illinois | IV |
| A095 | LD06-8970 | University of Illinois | IV |
| A096 | LD07-3823 | University of Illinois | IV |
| A097 | LD08-1719 | University of Illinois | IV |
| A098 | LD08-1935 | University of Illinois | IV |
| A099 | LD08-7227 | University of Illinois | IV |
| A100 | LD08-7916a | University of Illinois | IV |
| A101 | LS07-1343 | Southern Illinois University Carbondale | IV |
| A102 | LS07-1934 | Southern Illinois University Carbondale | IV |
| A103 | LS07-1942 | Southern Illinois University Carbondale | IV |
| A104 | LS07-2935 | Southern Illinois University Carbondale | IV |
| A105 | LS07-3125 | Southern Illinois University Carbondale | IV |
| A106 | LS07-3131 | Southern Illinois University Carbondale | IV |
| A107 | LS08-3120 | Southern Illinois University Carbondale | IV |
| A108 | LS08-4141 | Southern Illinois University Carbondale | IV |
| A109 | LS08-4348 | Southern Illinois University Carbondale | IV |
| A110 | LS08-4418 | Southern Illinois University Carbondale | IV |
| A111 | LS08-4542 | Southern Illinois University Carbondale | IV |
| A112 | LS08-4637 | Southern Illinois University Carbondale | IV |
| A113 | LS08-4934 | Southern Illinois University Carbondale | IV |
| A114 | LS08-4941 | Southern Illinois University Carbondale | IV |
| A115 | LS08-5515 | Southern Illinois University Carbondale | IV |
| A116 | LS08-5552 | Southern Illinois University Carbondale | IV |
| A117 | LS08-5837 | Southern Illinois University Carbondale | IV |
| A118 | LS08-5852 | Southern Illinois University Carbondale | IV |
| A119 | LS08-6003 | Southern Illinois University Carbondale | IV |
| A120 | LS08-6034 | Southern Illinois University Carbondale | IV |
| A121 | LS08-6332 | Southern Illinois University Carbondale | IV |
| A122 | SS07-18126 | University of Missouri | IV |
| A123 | SS07-18127 | University of Missouri | IV |
| A124 | SS07-18091 | University of Missouri | IV |
| A125 | SS07-16355 | University of Missouri | IV |
| A126 | SS08-3272 | University of Missouri | IV |
| A127 | SS08-3273 | University of Missouri | IV |
| A128 | R05-3239 | University of Arkansas | IV |
| A129 | R07-10231 | University of Arkansas | IV |
| A130 | R07-10244 | University of Arkansas | IV |
| A131 | UA 4805 | University of Arkansas | IV |
| A132 | UA 4910 | University of Arkansas | IV |
| A133 | M00-456052-2 | Minnesota Agricultural Experiment Station | I |
| A134 | Myc5171RR-2 | Susceptible check | I |
| A135 | AR11-114063 | Iowa State University | I |
| A136 | AR11-114057 | Iowa State University | I |
| A137 | AR11-114066 | Iowa State University | I |
| A138 | AR11-114059 | Iowa State University | I |
| A139 | AR11-114062 | Iowa State University | I |
| A140 | A09-755015 | Iowa State University | I |
| A141 | A10-453012 | Iowa State University | I |
| A142 | A10-453013 | Iowa State University | I |
| A143 | A10-453015 | Iowa State University | I |
| A144 | A10-455006 | Iowa State University | I |
| A145 | A10-456037 | Iowa State University | I |
| A146 | A10-456040 | Iowa State University | I |
| A147 | A10-457001 | Iowa State University | I |
| A148 | A10-457037 | Iowa State University | I |
| A149 | A10-556015 | Iowa State University | I |
| A150 | IA1024 | Iowa State University | I |
| A151 | IA1008 | Iowa State University | I |
| A152 | IA1008LF | Iowa State University | I |
| A153 | IA1018 | Iowa State University | I |
| A154 | M02-356043 | Minnesota Agricultural Experiment Station | I |
| A155 | M02-356123 | Minnesota Agricultural Experiment Station | I |
| A156 | M02-366027 | Minnesota Agricultural Experiment Station | I |
| A157 | M02-366130 | Minnesota Agricultural Experiment Station | I |
| A158 | M06-223004 | Minnesota Agricultural Experiment Station | I |
| A159 | M06-318015 | Minnesota Agricultural Experiment Station | I |
| A160 | M06-318018 | Minnesota Agricultural Experiment Station | I |
| A161 | M06-340092 | Minnesota Agricultural Experiment Station | I |
| A162 | M06-340100 | Minnesota Agricultural Experiment Station | I |
| A163 | M06-340120 | Minnesota Agricultural Experiment Station | I |
| A164 | M07-204016 | Minnesota Agricultural Experiment Station | I |
| A165 | M07-2134866 | Minnesota Agricultural Experiment Station | I |
| A166 | M05-297042 | Minnesota Agricultural Experiment Station | I |
| A167 | M05-307064 | Minnesota Agricultural Experiment Station | I |
| A168 | M05-319034 | Minnesota Agricultural Experiment Station | I |
| A169 | M06-101013 | Minnesota Agricultural Experiment Station | I |
| A170 | AR11SDS | Iowa State University | II |
| A171 | 233+RR | Resistance check | II |
| A172 | LD06-50113R-2 | University of Illinois | II |
| A173 | LD09-5393a | University of Illinois | II |
| A174 | LD09-6277 | University of Illinois | II |
| A175 | LD09-6408a | University of Illinois | II |
| A176 | LD09-13020a | University of Illinois | II |
| A177 | LD09-13026a | University of Illinois | II |
| A178 | LD09-13050a | University of Illinois | II |
| A179 | LD09-15175a | University of Illinois | II |
| A180 | LD09-15179a | University of Illinois | II |
| A181 | LD09-15195a | University of Illinois | II |
| A182 | LD09-16058 | University of Illinois | II |
| A183 | LD09-30015 | University of Illinois | II |
| A184 | LD09-30220 | University of Illinois | II |
| A185 | LD09-30224 | University of Illinois | II |
| A186 | AR09-291011 | Iowa State University | II |
| A187 | AR09-292004-2 | Iowa State University | II |
| A188 | AR08-286003 | Iowa State University | II |
| A189 | AR09-192019 | Iowa State University | II |
| A190 | E08005 | Michigan State University | II |
| A191 | E09088 | Michigan State University | II |
| A192 | 7P44 |  | II |
| A193 | IA2104 | Iowa State University | II |
| A194 | IA2076LF | Iowa State University | II |
| A195 | IA2099 | Iowa State University | II |
| A196 | IA2100 | Iowa State University | II |
| A197 | IA2101 | Iowa State University | II |
| A198 | IA2102 | Iowa State University | II |
| A199 | IA3042 | Iowa State University | II |
| A200 | IA3052 | Iowa State University | II |
| A201 | A10-454039 | Iowa State University | II |
| A202 | A10-554048 | Iowa State University | II |
| A203 | A10-555001 | Iowa State University | II |
| A204 | A10-555030 | Iowa State University | II |
| A205 | A10-653019 | Iowa State University | II |
| A206 | A10-655016 | Iowa State University | II |
| A207 | A10-558012 | Iowa State University | II |
| A208 | A10-656009 | Iowa State University | II |
| A209 | A10-656024 | Iowa State University | II |
| A210 | A10-656042 | Iowa State University | II |
| A211 | IA2092 | Iowa State University | II |
| A212 | IA2069 | Iowa State University | II |
| A213 | IA3051 | Iowa State University | II |
| A214 | IA2053 | Iowa State University | II |
| A215 | IA2040 | Iowa State University | II |
| A216 | IA2067 | Iowa State University | II |
| A217 | IA2096 | Iowa State University | II |
| A218 | IA2053LF | Iowa State University | II |
| A219 | IA2097 | Iowa State University | II |
| A220 | IA2079 | Iowa State University | II |
| A221 | IA2041 | Iowa State University | II |
| A222 | IA2103 | Iowa State University | II |
| A223 | The Ohio State#1 | The Ohio State University | II |
| A224 | The Ohio State#2 | The Ohio State University | II |
| A225 | The Ohio State#3 | The Ohio State University | II |
| A226 | The Ohio State#4 | The Ohio State University | II |
| A227 | The Ohio State#5 | The Ohio State University | II |
| A228 | The Ohio State#6 | The Ohio State University | II |
| A229 | AR03-361091-2 | Iowa State University | III |
| A230 | AR07-376031-2 | Iowa State University | III |
| A231 | LD08-923 | University of Illinois | III |
| A232 | LD08-RST5-10 | University of Illinois | III |
| A233 | LD09-3645 | University of Illinois | III |
| A234 | LD09-3913 | University of Illinois | III |
| A235 | LD09-9396 | University of Illinois | III |
| A236 | LD09-9476 | University of Illinois | III |
| A237 | LD09-10220 | University of Illinois | III |
| A238 | LD09-10242 | University of Illinois | III |
| A239 | LD09-10717 | University of Illinois | III |
| A240 | LD09-10911 | University of Illinois | III |
| A241 | LD09-11732 | University of Illinois | III |
| A242 | LD09-11822 | University of Illinois | III |
| A243 | AR11-314018 | Iowa State University | III |
| A244 | AR11-314016 | Iowa State University | III |
| A245 | AR11-314023 | Iowa State University | III |
| A246 | AR11-314015 | Iowa State University | III |
| A247 | IA3045 | Iowa State University | III |
| A248 | IA3027RA12 | Iowa State University | III |
| A249 | IA3045LF | Iowa State University | III |
| A250 | A10-454048 | Iowa State University | III |
| A251 | A10-654034 | Iowa State University | III |
| A252 | IA3023 | Iowa State University | III |
| A253 | IA3048 | Iowa State University | III |
| A254 | HS8W-3672 | The Ohio State University | III |
| A255 | HS8-3463 | The Ohio State University | III |
| A256 | HS8W-102 | The Ohio State University | III |
| A257 | HS8W-179 | The Ohio State University | III |
| A258 | WN0913499 | Syngenta | III |
| A259 | X2R3321 | Syngenta | III |
| A260 | X2R3422 | Syngenta | III |
| A261 | X2R3523 | Syngenta | III |
| A262 | X2R3524 | Syngenta | III |
| A263 | S37-B1 | Syngenta | III |
| A264 | SJ1012347 | Syngenta | III |
| A265 | LD07-3395bf | University of Illinois | IV |
| A266 | LD07-3395bl | University of Illinois | IV |
| A267 | LD07-3419 | University of Illinois | IV |
| A268 | LD08-7227bf | University of Illinois | IV |
| A269 | LD09-12184 | University of Illinois | IV |
| A270 | LD09-12449 | University of Illinois | IV |
| A271 | R05-4114 | University of Arkansas | IV |
| A272 | R07-1685 | University of Arkansas | IV |
| A273 | R08-141 | University of Arkansas | IV |
| A274 | R07-5351 | University of Arkansas | IV |
| A275 | R09-209 | University of Arkansas | IV |
| A276 | R09-430 | University of Arkansas | IV |
| A277 | R08-2797 | University of Arkansas | IV |
| A278 | R09-4571 | University of Arkansas | IV |
| A279 | R09-2567 | University of Arkansas | IV |
| A280 | R09-1589 | University of Arkansas | IV |
| A281 | R08-527 | University of Arkansas | IV |
| A282 | LS09-0333 | Southern Illinois University Carbondale | IV |
| A283 | LS09-0340 | Southern Illinois University Carbondale | IV |
| A284 | LS09-1021 | Southern Illinois University Carbondale | IV |
| A285 | LS09-1303 | Southern Illinois University Carbondale | IV |
| A286 | LS09-1527 | Southern Illinois University Carbondale | IV |
| A287 | LS09-1530 | Southern Illinois University Carbondale | IV |
| A288 | LS09-1803 | Southern Illinois University Carbondale | IV |
| A289 | LS09-2340 | Southern Illinois University Carbondale | IV |
| A290 | LS09-2342 | Southern Illinois University Carbondale | IV |
| A291 | LS09-2655 | Southern Illinois University Carbondale | IV |
| A292 | LS09-2659 | Southern Illinois University Carbondale | IV |
| A293 | LS09-2707 | Southern Illinois University Carbondale | IV |
| A294 | LS09-2722 | Southern Illinois University Carbondale | IV |
| A295 | LS09-5806 | Southern Illinois University Carbondale | IV |
| A296 | LS09-8104 | Southern Illinois University Carbondale | IV |
| A297 | IA4005 | Iowa State University | IV |
| A298 | X2R4220 | Syngenta | IV |
| A299 | HI1016812 | Syngenta | IV |
| A300 | BY1012348 | Syngenta | IV |
