# Supplementary material for: Comparison of Genetic Diversity between Chinese and American Soybean (Glycine max (L.)) Accessions Revealed by High-Density SNPs
Source: Front Plant Sci. 2017 Nov 30;8:2014. doi: 10.3389/fpls.2017.02014 (PMC5715234; doi:10.3389/fpls.2017.02014)
Supplement: Supplementary file 3 [file Table3.DOCX]

Supplementary Table S3 Assignment of the genotypes of the sub-groups (S Group) identified by STRUCTURE analysis based on the highest membership probability for single nucleotide polymorphism (SNP) markers.

| Structure group | Total | Origin | | Genetic distance within sub-group |
| --- | --- | --- | --- | --- |
|  |  | CN | US |  |
| S Group 1 | 273 | 257 | 16 | 0.2940 |
| S Group 2 | 304 | 20 | 284 | 0.2527 |

Note: CN is for China; and US is for United States of America.
