# Supplementary material for: Comparison of Genetic Diversity between Chinese and American Soybean (Glycine max (L.)) Accessions Revealed by High-Density SNPs
Source: Front Plant Sci. 2017 Nov 30;8:2014. doi: 10.3389/fpls.2017.02014 (PMC5715234; doi:10.3389/fpls.2017.02014)
Supplement: Supplementary file 4 [file Table4.DOCX]

Supplementary Table S4 The PCA data outputted from the software of TASSEL 5.0.

| PC1 | PC2 |
| --- | --- |
| 15.36975 | 8.264572 |
| 11.12242 | 13.58306 |
| 11.96186 | 14.02991 |
| 9.46947 | 5.859088 |
| 11.47221 | 12.52027 |
| 16.02767 | 13.16326 |
| 14.42222 | 13.16177 |
| -0.03722 | -8.97984 |
| 5.500971 | -1.22025 |
| 12.07753 | 10.39535 |
| 12.29715 | 10.75487 |
| 9.156512 | 3.279247 |
| 6.665608 | 4.253281 |
| 12.19911 | 5.16827 |
| 11.3264 | 17.20316 |
| 13.37239 | 13.22747 |
| 11.84066 | 3.019029 |
| 12.35095 | 13.34452 |
| 13.90825 | 1.866828 |
| 10.23175 | 2.191634 |
| 11.75656 | 11.26188 |
| 9.362679 | 12.08285 |
| 13.72154 | 8.234149 |
| 12.79809 | 5.905038 |
| 11.52799 | 4.487763 |
| 11.92694 | 7.433026 |
| 8.380399 | 15.61798 |
| 13.01708 | 17.20975 |
| 10.37258 | 11.38157 |
| 11.4414 | 6.873306 |
| 13.05862 | 9.583247 |
| 11.1755 | 14.46682 |
| 9.74323 | 11.13815 |
| 12.5308 | 1.775646 |
| 8.21083 | 8.219442 |
| 11.88411 | 17.80004 |
| 11.54845 | 6.577662 |
| 12.81445 | 0.646933 |
| 9.491734 | 10.95186 |
| 14.27598 | 8.578741 |
| 11.46692 | 1.37908 |
| 10.89807 | 0.116413 |
| 10.4269 | 4.135598 |
| 11.20425 | 14.46308 |
| 10.52128 | 13.28008 |
| 11.12661 | 8.641789 |
| 13.16504 | 6.607341 |
| 13.25957 | 6.375666 |
| 13.48242 | -2.69784 |
| 12.55309 | 11.13163 |
| 10.77765 | -3.24886 |
| 4.041077 | 5.308393 |
| 11.77489 | -6.80885 |
| 4.672329 | 6.594026 |
| 14.97395 | 13.91116 |
| 9.342359 | 9.701327 |
| 9.318112 | 2.225264 |
| 9.377108 | 8.151655 |
| 10.71055 | 10.50612 |
| 11.13688 | 8.375368 |
| 9.637041 | 16.14403 |
| 8.992576 | 8.105422 |
| 11.93442 | 15.91643 |
| 12.80391 | 13.78559 |
| 11.853 | 11.26748 |
| 9.645571 | 16.12677 |
| 11.45478 | -3.21319 |
| 8.408959 | 8.107605 |
| 11.42281 | 15.25349 |
| 9.453595 | 11.39319 |
| 12.17031 | 5.315425 |
| 6.463152 | 5.713601 |
| 4.664387 | 5.487222 |
| 8.245144 | 4.757064 |
| 12.54813 | 13.68472 |
| 10.72486 | 9.361819 |
| 10.63016 | 14.29745 |
| 11.44507 | -8.42522 |
| -4.23937 | -0.91614 |
| 12.1984 | 11.54148 |
| 12.1984 | 11.56809 |
| 10.83226 | 10.41978 |
| 11.09237 | 3.827698 |
| 12.6838 | -3.53397 |
| 10.73746 | -2.12289 |
| 4.300202 | 1.357063 |
| 14.16333 | 0.244949 |
| 13.14649 | -6.74597 |
| 11.05048 | -3.30306 |
| 9.88932 | -3.94849 |
| 11.83943 | -7.73157 |
| 13.55639 | -4.75604 |
| 16.08863 | -3.21215 |
| 14.28749 | -5.55367 |
| 1.711626 | -0.39024 |
| 13.12037 | 3.906674 |
| 8.272841 | 2.961473 |
| 14.53451 | -6.01016 |
| 16.09401 | -6.56596 |
| 13.22639 | 11.03266 |
| 11.9537 | -9.00008 |
| 7.10946 | 0.254767 |
| 14.76408 | -7.60272 |
| 14.70897 | -4.36325 |
| 17.96415 | -1.05108 |
| 17.57201 | -9.00122 |
| 16.57761 | -1.57313 |
| 14.78842 | -10.0757 |
| 10.99117 | -4.62311 |
| 17.60062 | -9.05401 |
| 11.1194 | -6.85142 |
| 12.45543 | -10.2122 |
| 13.4581 | 0.084737 |
| 16.52973 | -3.96937 |
| 8.300034 | -10.6947 |
| 13.27487 | -7.24928 |
| 14.88804 | -1.59986 |
| 13.41561 | -4.81469 |
| 5.444264 | -3.39697 |
| 10.34443 | -9.2353 |
| 15.30833 | -8.3116 |
| 9.631517 | 0.694265 |
| 18.1181 | -8.92571 |
| 14.62082 | -7.51285 |
| 10.7992 | -4.94526 |
| 14.26809 | -9.66516 |
| 17.00133 | -9.19831 |
| 9.385446 | 7.927515 |
| 12.36119 | -8.71767 |
| 13.54031 | -7.69356 |
| 14.43066 | -3.10039 |
| 12.10784 | -3.99071 |
| 13.42392 | -4.38658 |
| 16.97277 | -8.72385 |
| 9.820688 | -10.4683 |
| 16.28283 | -2.64748 |
| 16.00461 | -6.24907 |
| 14.15213 | -9.09776 |
| 13.75043 | 8.042954 |
| 12.76288 | -7.98773 |
| 11.62833 | -5.57315 |
| 11.67428 | -9.44816 |
| 10.51806 | 13.26033 |
| 12.75492 | 0.047075 |
| 15.41397 | 6.29109 |
| 15.50957 | -2.74769 |
| 9.67516 | -4.96665 |
| 14.6666 | -6.71827 |
| 8.440384 | -4.01792 |
| 9.652137 | -3.5528 |
| 9.854844 | -3.24049 |
| 1.152742 | 3.190321 |
| -14.6696 | -3.12876 |
| 4.92378 | -2.27429 |
| 5.445868 | -7.81886 |
| 3.710537 | -1.86449 |
| -1.2258 | -2.12332 |
| -13.8066 | -3.21968 |
| 2.254646 | 2.279928 |
| 2.195768 | 8.37317 |
| -3.96195 | -2.8626 |
| -3.69439 | -0.6551 |
| -1.73636 | -1.19546 |
| 1.050029 | 3.865456 |
| -15.9291 | -2.63295 |
| -4.47967 | 2.049498 |
| 8.855554 | 2.114405 |
| 9.253059 | -3.7958 |
| 3.643795 | 6.078781 |
| 3.696162 | -1.35733 |
| -6.10429 | 3.059275 |
| 5.518826 | 0.288169 |
| 1.232741 | -1.59875 |
| 10.57072 | -2.74401 |
| -6.18488 | -0.9182 |
| -2.25522 | 3.209166 |
| 3.409243 | -4.25886 |
| 4.043587 | -4.45341 |
| 4.377162 | -5.38526 |
| 0.361236 | 0.336378 |
| 5.705169 | -1.77483 |
| 1.003695 | 3.683165 |
| 5.570236 | 4.017815 |
| 6.523494 | -3.61126 |
| -14.6715 | -3.15289 |
| 0.126126 | 1.123943 |
| 6.630084 | -5.17603 |
| 8.483248 | 1.654876 |
| 6.342085 | -5.2115 |
| 8.101302 | -5.42938 |
| -1.13683 | -3.49373 |
| -1.07508 | -0.21148 |
| -4.90369 | -0.32614 |
| -0.18574 | 2.733876 |
| 11.48192 | 7.824786 |
| 16.03028 | 13.55776 |
| 7.851646 | 5.691834 |
| 11.80503 | 7.309958 |
| 11.07889 | -0.67758 |
| -5.99711 | 1.004527 |
| 10.31926 | 7.465169 |
| 7.828746 | -4.15905 |
| 8.425825 | 6.927095 |
| 15.11977 | -7.11241 |
| 9.786289 | 0.450662 |
| 7.174923 | -5.79921 |
| 12.18491 | -4.7971 |
| 13.44332 | -2.85379 |
| 14.78215 | -7.69175 |
| 13.95193 | -8.3614 |
| 10.37051 | -9.62604 |
| 10.10581 | -2.73699 |
| 12.65172 | -7.52643 |
| 10.61401 | -11.0791 |
| 10.21538 | -9.97699 |
| 10.89508 | -3.486 |
| 8.845372 | 0.759186 |
| 9.9056 | -4.81313 |
| 12.13753 | -9.10621 |
| 10.78595 | -5.31638 |
| 14.05003 | -10.8695 |
| 11.87569 | -7.05617 |
| 10.34012 | -9.35636 |
| 11.24028 | -0.33983 |
| 13.56118 | -0.28726 |
| 13.96409 | -3.89565 |
| 9.369902 | 4.488616 |
| 9.108166 | -3.41278 |
| 13.78015 | 1.730014 |
| 14.45537 | -1.51777 |
| 13.48714 | -4.76187 |
| 11.7827 | 0.744435 |
| 11.369 | -10.559 |
| 9.994155 | 5.501307 |
| 14.76848 | -2.09781 |
| 7.172083 | 2.008395 |
| 8.30997 | -2.6601 |
| 7.251021 | -10.1076 |
| 12.54872 | -2.96466 |
| 7.727957 | 3.392671 |
| 5.787005 | 1.224892 |
| 9.865923 | -4.89354 |
| 10.26836 | -7.2193 |
| 11.54965 | -6.22678 |
| 13.77939 | -6.5677 |
| 13.87179 | -4.22376 |
| 10.26541 | -10.2474 |
| 8.286834 | -3.42044 |
| 9.449749 | -3.34516 |
| 12.02559 | -4.61082 |
| 14.67594 | -7.35205 |
| 9.534599 | -4.80832 |
| 11.2985 | -5.73685 |
| 9.467843 | -2.9892 |
| 10.57484 | -7.34584 |
| 9.974758 | -1.12875 |
| 10.3893 | -7.77605 |
| 12.79536 | -8.60475 |
| 12.42027 | -7.42312 |
| 12.71205 | -7.99926 |
| 17.46725 | -9.12642 |
| 12.4811 | -8.06884 |
| 11.01954 | -7.53583 |
| 11.37097 | -6.05941 |
| 7.12721 | -2.30677 |
| 2.845664 | 0.367047 |
| 12.98798 | -0.53375 |
| 12.77319 | -8.18448 |
| 13.19478 | -7.89156 |
| 6.900514 | -11.5861 |
| 9.709819 | 1.457043 |
| 7.942937 | -2.27506 |
| -10.1456 | -3.16117 |
| 8.693901 | 0.559793 |
| 1.263751 | 2.09177 |
| -1.97779 | 1.169145 |
| 7.615207 | 2.24336 |
| -2.84491 | -9.24238 |
| -2.14046 | -2.20258 |
| -7.26751 | -1.17683 |
| -8.98652 | 0.384664 |
| -14.4437 | 2.163023 |
| -15.099 | 0.362189 |
| -8.71217 | -5.91388 |
| -6.99714 | -1.04773 |
| -7.2644 | -1.97744 |
| 0.761332 | -2.48336 |
| -1.23743 | -5.32004 |
| -4.06909 | -1.08678 |
| -5.27968 | -3.25867 |
| -0.78706 | -5.53675 |
| 2.61452 | -1.78619 |
| -2.7087 | 7.189495 |
| -2.78996 | -2.69812 |
| -12.5636 | -0.69254 |
| -8.46705 | 1.561403 |
| -12.1802 | 4.445477 |
| -10.5725 | -4.05392 |
| -8.9976 | 5.225308 |
| -12.6361 | -3.25971 |
| -10.9192 | -2.40974 |
| 2.888531 | -6.74448 |
| 8.351534 | 10.35682 |
| 2.600591 | -6.61406 |
| -10.4951 | 2.069506 |
| 1.134604 | -1.92635 |
| -6.17958 | -2.9747 |
| -7.91115 | -2.21119 |
| -9.34463 | -0.26931 |
| -12.7553 | 9.696603 |
| -6.89414 | 3.331438 |
| -12.6088 | 8.883634 |
| -12.1082 | 10.09537 |
| -12.1613 | 10.2973 |
| -12.6246 | 10.05522 |
| -12.1112 | 10.14615 |
| -12.6775 | 9.867569 |
| -12.1437 | 9.966455 |
| -11.7977 | -0.50488 |
| -11.9268 | 4.401792 |
| -9.63416 | 5.794418 |
| -15.4823 | 4.19649 |
| -15.8168 | -3.89956 |
| -11.6426 | 1.038029 |
| -16.3139 | -1.33802 |
| -11.4592 | 3.868519 |
| -8.8652 | 2.407771 |
| -14.4805 | 2.0061 |
| -5.66924 | -1.70789 |
| -7.97124 | -3.34327 |
| -11.7737 | -2.86466 |
| -11.3093 | -3.4847 |
| -11.3032 | -1.21077 |
| -8.68252 | 1.066896 |
| -8.54078 | -2.48892 |
| -13.4379 | 9.626894 |
| -12.7884 | 9.333826 |
| -11.9432 | -0.11413 |
| -10.1282 | 1.827446 |
| -12.3462 | 5.322707 |
| -12.6494 | 0.632945 |
| -14.3429 | 4.895584 |
| -11.7316 | -1.59123 |
| -12.3627 | 0.114442 |
| -13.2451 | 0.10315 |
| -8.24328 | -6.38567 |
| -9.53799 | -1.47783 |
| -8.5908 | 2.718971 |
| -8.00275 | -3.53838 |
| -8.36638 | 1.70921 |
| -9.80546 | 0.099707 |
| -12.5544 | 1.112927 |
| -14.5369 | -4.14104 |
| -12.9653 | -4.26549 |
| -12.9954 | -4.28363 |
| -13.3004 | -1.59169 |
| -7.91767 | -0.91543 |
| -7.56958 | -1.40245 |
| -5.96964 | 5.546803 |
| -11.6292 | 1.705039 |
| -13.056 | -0.69849 |
| -13.543 | 2.757935 |
| -9.94279 | -1.52477 |
| -9.32323 | -0.00573 |
| -4.28022 | -8.01677 |
| 3.634119 | -5.53312 |
| 3.032093 | 3.110103 |
| -7.89982 | -0.43839 |
| -2.57532 | -6.1346 |
| -13.5892 | 1.175086 |
| -14.9119 | 3.45634 |
| -10.2617 | -2.6757 |
| -11.6999 | 1.275021 |
| -13.5568 | 3.577394 |
| -3.19422 | -0.89218 |
| -7.63229 | -3.40485 |
| -14.2765 | -0.62947 |
| -11.612 | 6.412945 |
| -3.65048 | -2.6077 |
| -8.30353 | 0.798505 |
| -11.5535 | 1.111611 |
| -13.6925 | 0.106357 |
| -13.0234 | -3.66298 |
| -10.4946 | -0.55555 |
| -15.8329 | -2.75834 |
| -7.40313 | -1.56743 |
| -14.0085 | 3.221669 |
| -12.5281 | -3.76613 |
| -9.32354 | 0.21412 |
| -11.3664 | -0.33528 |
| -15.0448 | 1.35088 |
| -11.5501 | -2.77489 |
| -12.0735 | 2.060497 |
| -15.0422 | -2.41825 |
| -12.0591 | 0.395207 |
| -10.6452 | -3.40137 |
| -6.66227 | -4.73635 |
| -1.19876 | 2.847763 |
| -5.80281 | 5.781147 |
| -4.59982 | 2.583835 |
| -6.67018 | 1.854464 |
| -3.26518 | -2.68604 |
| -12.7856 | 2.382839 |
| -13.4152 | 2.375762 |
| 4.685086 | 1.794658 |
| -2.55408 | 7.741375 |
| -2.57746 | 7.751479 |
| -4.34981 | 3.904096 |
| -9.54917 | 0.913228 |
| -2.12737 | -2.27519 |
| -15.1857 | 0.58628 |
| -5.09249 | -3.71966 |
| -8.38338 | -4.12631 |
| -3.06418 | -1.99847 |
| 2.30193 | 0.723391 |
| -12.0506 | -0.34026 |
| -13.3223 | -2.04903 |
| -12.662 | 0.938945 |
| -10.8606 | -1.52231 |
| 2.094265 | 0.617763 |
| 2.378801 | 0.667437 |
| -9.02947 | -7.00523 |
| -9.16295 | -2.93317 |
| -12.2439 | -4.27396 |
| -10.2625 | -2.46894 |
| 2.096367 | 0.695198 |
| -12.6482 | -5.53527 |
| -5.69092 | -2.33921 |
| -5.34054 | -1.79833 |
| -1.21044 | -4.30359 |
| -5.64422 | -4.97174 |
| 2.131851 | 0.595457 |
| -4.99621 | -2.22021 |
| -6.78463 | -4.58123 |
| -4.19526 | -0.82196 |
| -3.93194 | -4.31177 |
| -0.48628 | -1.46263 |
| -5.07738 | 4.550944 |
| -9.47264 | 4.78617 |
| -6.93211 | 4.677399 |
| -3.66684 | 3.863165 |
| -2.67926 | -2.40687 |
| -0.71901 | -3.68823 |
| -1.51076 | -5.43437 |
| -0.24812 | -6.57155 |
| -3.61645 | -4.33545 |
| -2.80712 | -2.61569 |
| -12.4056 | -0.37382 |
| -10.0184 | 5.471106 |
| -6.38264 | -7.15612 |
| -8.40029 | -1.88228 |
| -10.7765 | 1.517616 |
| -13.0827 | 0.597784 |
| -13.0101 | 4.421363 |
| -11.9237 | 2.316667 |
| -13.7092 | 9.81088 |
| -13.6842 | 9.866976 |
| -13.6621 | 9.98328 |
| -11.7544 | -3.67053 |
| -11.2973 | 7.671721 |
| -8.4088 | -0.03387 |
| -10.3369 | -1.34154 |
| -6.98084 | 1.215404 |
| -6.79894 | -1.0197 |
| -11.2937 | 3.905107 |
| -11.5704 | 3.134407 |
| -10.1147 | 1.869163 |
| -9.87773 | -0.5326 |
| 3.14264 | -0.46813 |
| -5.68357 | -5.16881 |
| -6.47203 | -6.48223 |
| -12.1242 | 0.696004 |
| -14.2414 | -3.03791 |
| -7.39222 | -2.75028 |
| -9.8651 | -4.41367 |
| -8.85308 | -2.28534 |
| -13.6856 | 2.750089 |
| -12.1113 | -2.9526 |
| -8.82113 | -1.85031 |
| -12.3604 | -3.43763 |
| -13.4033 | -0.70579 |
| -9.35653 | -0.27145 |
| -13.3005 | -2.27939 |
| -11.9993 | -3.60847 |
| -8.17899 | -2.99879 |
| -10.3789 | -2.37028 |
| -9.0719 | -4.45346 |
| -9.29467 | -0.98379 |
| -8.21949 | -4.30382 |
| -8.3989 | -2.83542 |
| -5.91986 | -5.97649 |
| -5.51916 | -6.01041 |
| 2.000148 | -6.16463 |
| -4.95987 | -3.93913 |
| -5.59138 | -4.27325 |
| -13.3463 | 3.077642 |
| -9.88706 | -2.90986 |
| -5.38507 | -1.46451 |
| -5.53474 | -5.31941 |
| -12.3134 | -5.39385 |
| -16.004 | 2.605413 |
| -12.2388 | 3.788127 |
| -11.6923 | 4.005232 |
| -11.6485 | 1.187394 |
| -16.4253 | -1.3016 |
| -7.66869 | -3.27782 |
| -11.7917 | -2.90528 |
| -12.2742 | 2.757411 |
| -14.5851 | -0.52624 |
| -12.3955 | 0.604021 |
| -12.0514 | -0.42256 |
| -13.8776 | 2.836685 |
| -13.7218 | -2.81627 |
| -10.0946 | -6.62159 |
| -9.64972 | 0.32707 |
| -10.1837 | 2.227669 |
| -13.5069 | 8.086743 |
| -14.6986 | -0.1909 |
| -13.4161 | 8.583988 |
| -7.8126 | -8.55834 |
| -6.84348 | -5.40814 |
| -5.4574 | -4.34147 |
| -6.08816 | -7.53979 |
| -6.85425 | -5.14638 |
| -7.1518 | -3.84787 |
| -6.83657 | -5.17704 |
| -10.8306 | -1.42228 |
| -9.78342 | -2.24436 |
| -15.4407 | 1.000675 |
| -12.364 | 0.454551 |
| -9.15219 | -1.8663 |
| -5.55261 | -3.50172 |
| -4.48912 | -0.50519 |
| -8.33274 | -5.29686 |
| -8.40424 | -1.34995 |
| -13.5876 | -1.04385 |
| -14.1733 | 0.334521 |
| -14.0478 | 0.057108 |
| -14.0798 | 0.032313 |
| -6.41352 | -3.47544 |
| -8.56936 | -4.85162 |
| -14.0089 | -2.65332 |
| -14.5261 | -2.90596 |
| -11.8243 | -4.85994 |
| -7.14985 | -0.99451 |
| -11.3879 | -5.44874 |
| -11.1144 | 4.860848 |
| -8.96284 | 4.703831 |
| -5.79563 | 3.557334 |
| -1.96982 | -1.90062 |
| -0.16541 | -0.85704 |
| -0.63848 | 0.369981 |
| -5.35678 | 5.346935 |
| -2.36609 | -2.20166 |
| -3.54321 | -3.91907 |
| -2.91799 | 2.427806 |
| -3.56065 | -2.11802 |
| -2.83049 | 6.664625 |
| 0.595379 | -1.83032 |
| -5.93318 | 1.81724 |
| -8.57455 | 7.337393 |
| -8.99678 | 2.677751 |
| -9.73695 | -2.77298 |
| -13.8363 | 0.340244 |
| -10.3597 | 1.468769 |
| -10.9279 | 0.870268 |
| -14.4067 | -0.63235 |
| -10.8425 | 0.395697 |
| -12.132 | 0.424268 |
| -11.5306 | 4.606278 |
| -11.5975 | 2.126044 |
| -11.557 | -2.34631 |
| -2.61226 | 0.249917 |
| -13.3793 | 1.082614 |
| -14.8725 | -4.98877 |
| -6.02471 | 0.094179 |
| -15.0131 | -2.59512 |

PC 1 and PC 2 are the first and second principal coordinate, respectively.
