# Supplementary material for: Comparison of Genetic Diversity between Chinese and American Soybean (Glycine max (L.)) Accessions Revealed by High-Density SNPs
Source: Front Plant Sci. 2017 Nov 30;8:2014. doi: 10.3389/fpls.2017.02014 (PMC5715234; doi:10.3389/fpls.2017.02014)
Supplement: Supplementary file 5 [file Table5.DOCX]

Supplementary Table S5 Analysis of molecular variance (AMOVA) and *F_ST_* for the two pre-defined subpopulations.

| Source of variation | Degree of freedom | Sum of squares | Variance components | Percentage of variance components |
| --- | --- | --- | --- | --- |
| Among populations | 1 | 89319.05 | 153.93 | 19.33 |
| Within populations | 1152 | 740197.38 | 642.53 | 80.67 |
| Total | 1153 | 829516.44 | 796.46 |  |

Population pair-wise *F_ST_*: 0.1933 (*P*<0.01).
