# Supplementary material for: Comparison of Genetic Diversity between Chinese and American Soybean (Glycine max (L.)) Accessions Revealed by High-Density SNPs
Source: Front Plant Sci. 2017 Nov 30;8:2014. doi: 10.3389/fpls.2017.02014 (PMC5715234; doi:10.3389/fpls.2017.02014)
Supplement: Supplementary file 6 [file Table6.DOCX]

Supplementary Table S6 Genetic distance and relative kinships revealed by the 5,195 polymorphic SNP markers in soybean accessions of the diversity panel.

|  | Genetic distance | Relative kinship |
| --- | --- | --- |
| CN | 0.3223 (0～0.5815) | 0.0477 (0～1.4108) |
| US | 0.2932 (0～0.5204) | 0.0416 (0～1.5637) |
| CN+US | 0.3794 (0.1225～0.5899) | 0.0195 (0～1.0204) |

Note: CN is for China; and US is for United States of America.
